# Single-Molecule Detection of α-Synuclein Oligomers in Parkinson’s Disease Patients Using Nanopores

**DOI:** 10.1021/acsnano.3c08456

**Published:** 2023-11-10

**Authors:** Yaxian Liu, Xiaoyi Wang, Giulia Campolo, Xiangyu Teng, Liming Ying, Joshua B. Edel, Aleksandar P. Ivanov

**Affiliations:** † Department of Chemistry, Imperial College London, Molecular Sciences Research Hub, London W12 0BZ, United Kingdom; ‡ National Heart and Lung Institute, Imperial College London, Molecular Sciences Research Hub, London W12 0BZ, United Kingdom

**Keywords:** single-molecule, nanopore, carrier, α-Synuclein oligomers, protein aggregation, cerebrospinal fluid, Parkinson’s disease, neurodegeneration

## Abstract

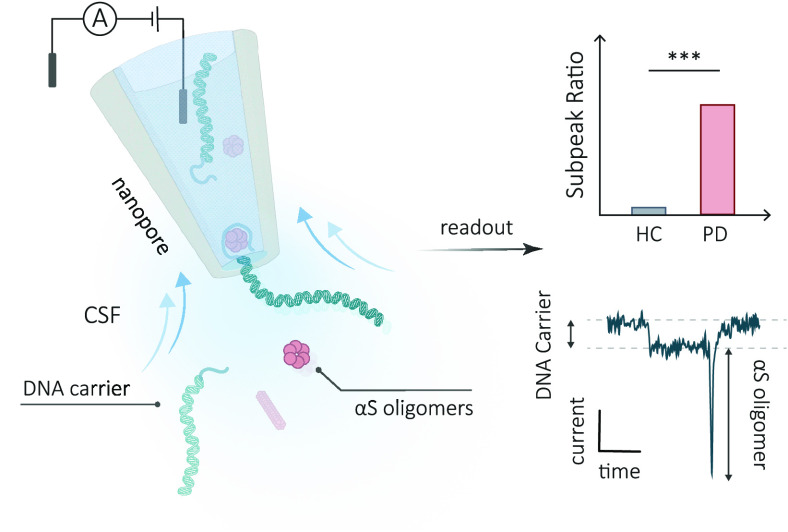

α-Synuclein (α-Syn) is an intrinsically disordered protein whose aggregation in the brain has been significantly implicated in Parkinson’s disease (PD). Beyond the brain, oligomers of α-Synuclein are also found in cerebrospinal fluid (CSF) and blood, where the analysis of these aggregates may provide diagnostic routes and enable a better understanding of disease mechanisms. However, detecting α-Syn in CSF and blood is challenging due to its heterogeneous protein size and shape, and low abundance in clinical samples. Nanopore technology offers a promising route for the detection of single proteins in solution; however, the method often lacks the necessary selectivity in complex biofluids, where multiple background biomolecules are present. We address these limitations by developing a strategy that combines nanopore-based sensing with molecular carriers that can specifically capture α-Syn oligomers with sizes of less than 20 nm. We demonstrate that α-Synuclein oligomers can be detected directly in clinical samples, with minimal sample processing, by their ion current characteristics and successfully utilize this technology to differentiate cohorts of PD patients from healthy controls. The measurements indicate that detecting α-Syn oligomers present in CSF may potentially provide valuable insights into the progression and monitoring of Parkinson’s disease.

## Introduction

Parkinson’s disease (PD) has been considered the second most common neurodegenerative disease, with clinical symptoms including bradykinesia, rigidity, rest tremor, and postural disturbance, severely impacting the aging population worldwide.^1^ The current diagnosis of PD heavily relies on the clinical history and the manifestation of symptoms, which can often go unnoticed for extended periods, hindering the potential diagnosis at early stages and making it hysteretic and inaccurate.^2−4^ The hallmark of PD is the loss of the dopaminergic neurons in the substantia nigra pars compacta and the presence of Lewy bodies,^4−6^ primarily composed of insoluble α-Synuclein (α-Syn) aggregates as inclusions. α-Syn (14.5 kDa, pI of 4.7) is a small neuronal protein abundant in the brain,^7^ responsible for regulating synaptic vesicle trafficking and subsequent neurotransmitter release.^8^ Misfolding of monomeric α-Syn into soluble oligomers has been linked to high cytotoxicity and the induction of axonal dysfunction;^2,9,10^ however, the molecular basis of this toxicity remains yet to be established.^11^ The accumulation of toxic α-Syn oligomers in CSF or plasma has been significantly related to the onset and progression of neurodegenerative synucleinopathies, indicating the potential of α-Syn oligomers as a biomarker candidate.^12−14^ Currently, identification and quantification of α-Syn typically rely on enzyme-linked immunosorbent assay (ELISA)^15,16^ and fluorescence-based microscopy.^14,16−18^ Based on ELISA measurements, it has been found that CSF from healthy individuals and PD patients contains comparable levels of total α-Syn in the range of 0.5–8 ng mL^–1^.^19^ In contrast, the concentrations of α-Syn oligomers are notably lower, 50–100 pg mL^–1^ in non-PD patients,^20^ while PD patients tend to exhibit a significant increase in the concentration of α-Syn oligomers compared to healthy individuals.^20−22^ However, direct detection of α-Syn oligomers from clinical samples poses significant challenges due to their low concentration in bodily fluids such as CSF,^20,23,24^ their heterogeneity in size and shape, their transient nature during protein turnover, and the presence of similar structures with other amyloid proteins.^2^

Recent advances in single-molecule imaging techniques, specifically those based on single-molecule confocal fluorescence, have addressed some of the detection limitations and demonstrated the potential diagnostic value of performing single-molecule detection of oligomers in the CSF and serum of PD patients. Using aptamer DNA-PAINT and single-aggregate confocal fluorescence, it is possible to image single α-Syn aggregates ranging in size typically from 20 to 200 nm.^13^ However, this method cannot recognize oligomers smaller than 20 nm, which may be highly cytotoxic as smaller aggregates can permeate lipid membranes more easily and induce more severe inflammation compared to the larger aggregates.^10^ One promising technology for detecting small oligomeric species *in vitro* involves the utilization of nanopores for studying unlabeled analytes at the single-molecule level.^25−27^ In particular, engineered nanopore interfaces offer high sensitivity to the molecular properties of analytes that can enable detection within complex samples.^28^ Similar approaches have been used in amyloid studies for measuring protein aggregates and tracking the aggregation dynamics.^29−35^ However, to date, nanopore sensing of amyloid proteins has only been performed in artificial buffers with a high protein concentration, deviating from the conditions present in real clinical samples.^30−33,36,37^ Furthermore, the lack of selectivity presents considerable difficulty in differentiating the target protein from multiple analytes with similar sizes and shapes, as nanopore measurements generally provide volumetric information on the proteins being analyzed.

**Figure 1:**
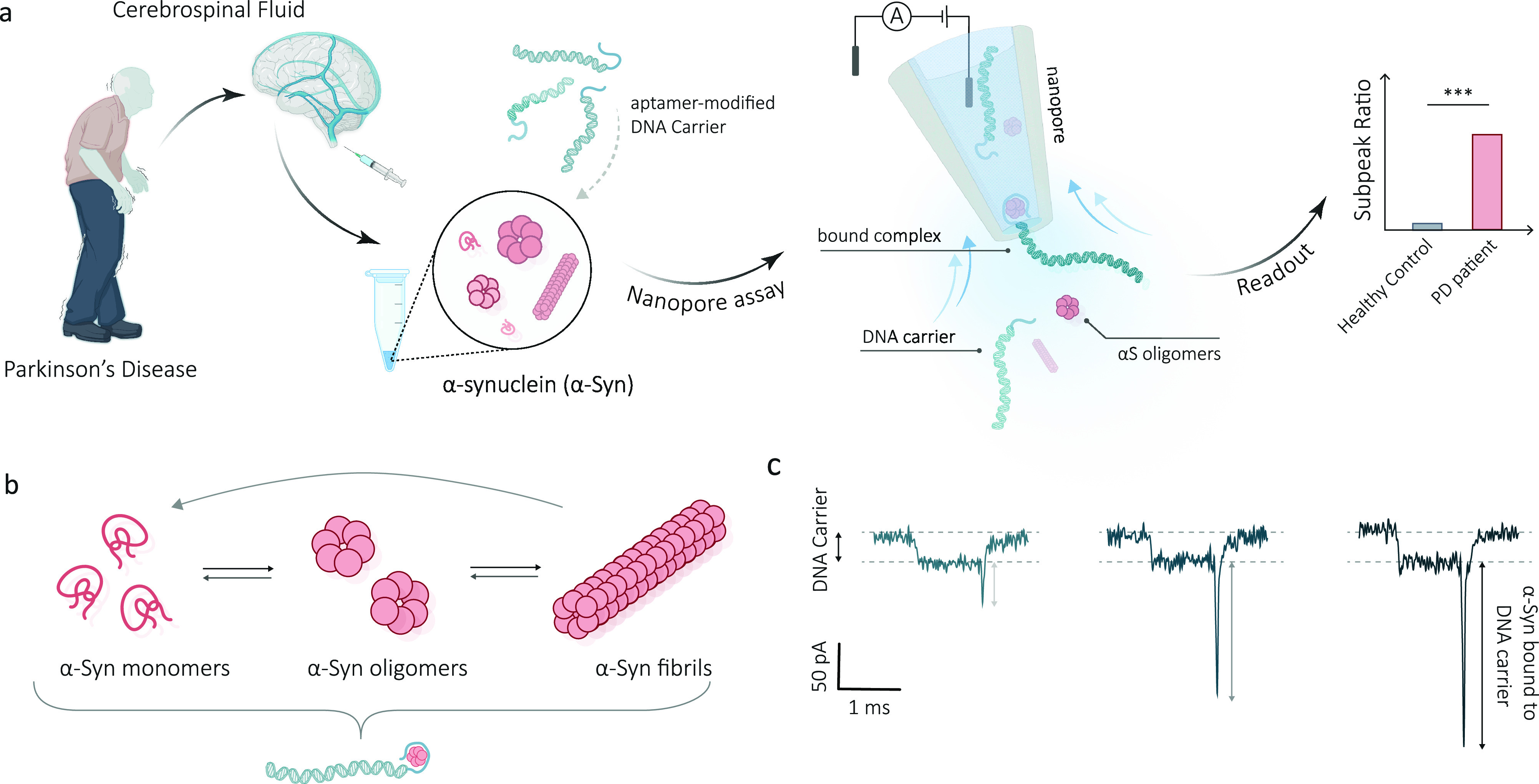
Schematic illustration of the workflow for the detection of α-Syn oligomers in CSF. (a) α-Syn oligomers in CSF are captured by DNA carriers modified with aptamers, forming a complex between the carrier and the protein. These DNA-bound α-Syn oligomer complexes are then translocated through a nanopore, where they can be recognized by a distinct subpeak signal. The subpeak ratio, indicating the proportion of signals with subpeaks relative to the total signal count, is indicative of the concentration of oligomeric forms in the clinical sample. (b) Schematic of α-Syn aggregation, starting with monomers assembling into oligomers and progressing to fibril formation. (c) Typical signals generated by different-sized α-Syn oligomers bound to carriers. When the carrier is translocated through the nanopore, a characteristic reduction in the nanopore current is observed. The binding of α-Syn oligomers to aptamer-modified carriers results in distinctive subpeaks superimposed on the DNA carrier-only current signal. The subpeak current amplitude is correlated to the size of α-Syn aggregates.

To address these limitations, we present an approach that employs an aptamer-modified DNA carrier to selectively capture and detect soluble α-Syn oligomers directly in clinical samples by using a quartz nanopore (Figure 1a). DNA molecular carriers are particularly well-suited for protein sensing primarily due to their long molecular length and charged backbone, facilitating easier protein transport and detection.^38^ Furthermore, DNA carriers can be readily engineered to incorporate specific protein recognition sites, such as aptamers,^39−41^ which substantially tackle the selectivity issue of nanopore detection. Using an aptamer selective to soluble α-Syn oligomers, we could quantify the size distribution and relative concentration of the α-Syn oligomeric species. Our approach shows promising compatibility for direct implementation in clinical biofluids such as CSF, using initial sample volumes (1.5 μL) substantially lower than that in conventional ELISA assays.^15^ With this method, we can differentiate cohorts of PD patients and healthy controls by assessing their α-Syn oligomer distribution in CSF. Performing such detection at the single-molecule level highlights the future promise of using a nanopore-based strategy for patient stratification and for the potential monitoring of disease progression and evaluating treatment efficacy.

## Results and Discussion

### Nanopore Measurements and Carrier Design

Nanopipettes were fabricated using laser-assisted pulling of quartz capillaries, as previously described, resulting in the formation of nanopores at the very tip region.^41,42^ The average size of the nanopores was determined to be 22 ± 3 nm through scanning electron microscopy (SEM) and ionic current conductance measurements (Figures S1 and S2). An Ag/AgCl quasi-reference counter electrode (QRCE) was inserted in the outside bath, and the patch Ag/AgCl electrode was inside the nanopipette. Carriers and protein analytes were introduced into the bath. Under such conditions, carriers were captured and transported from the outside of the nanopipette to the inside at a positive bias of +300 mV vs Ag/AgCl QRCE. To optimize the signal-to-noise ratio (SNR) of the nanopore measurements and regulate the translocation speed of DNA carriers, we employed relatively high ion strengths (2 M LiCl in Tris-EDTA pH 8.0 buffer).^43^

**Figure 2:**
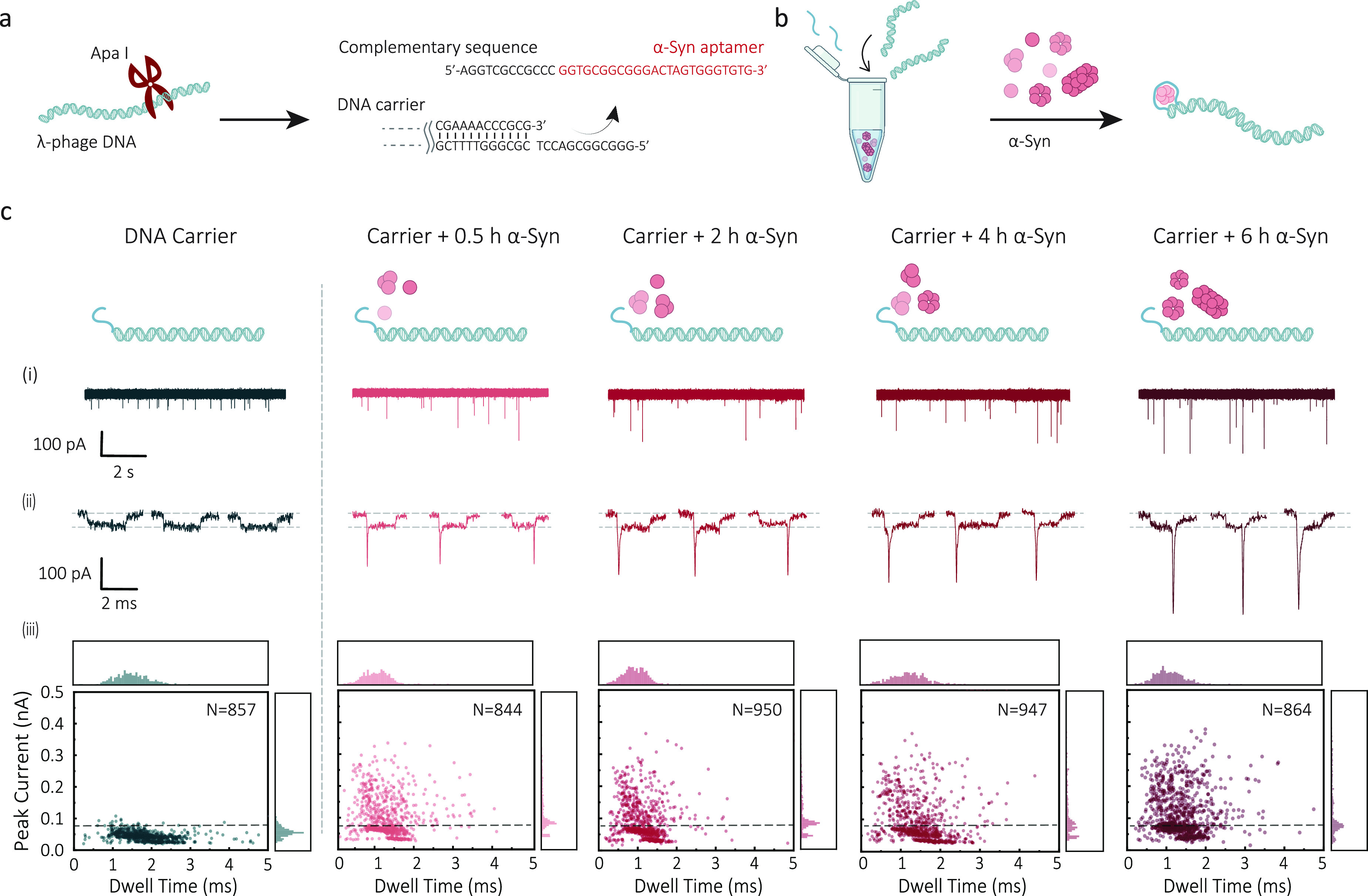
Nanopore detection of α-Syn oligomers using carriers. (a) Schematic of the carrier preparation. A 10 kbp dsDNA was digested from λ DNA, leaving a 12-base overhang at either end. These ends hybridized to a complementary strand that incorporates a 24-base α-Syn aptamer, resulting in the formation of a DNA carrier. (b) Schematic for preparing the α-Syn aptamer modified carrier bound to α-Syn oligomers. (c) Statistics of carrier bound to different α-Syn oligomers (0.5 h, 2 h, 4 and 6 h aggregation). (i) Typical current–time traces for each respective sample. The peak current progressively increases as the aggregation time of α-Syn oligomers extends. (ii) Three representative signals of each sample. (iii) Scatter plots and corresponding histogram of peak current versus dwell time. A dashed line indicates the threshold for differentiating between carrier only and α-Syn oligomers bound carrier. All experiments were performed in 2 M LiCl at +300 mV and repeated using a minimum of three nanopipettes.

Double-stranded DNA has been demonstrated as an ideal molecular carrier for protein nanopore sensing due to its rigid structure, advanced DNA technology, and reproducible nanopore readouts.^39^ In this work, we utilized a 10 kbp dsDNA fragment as the carrier obtained by cutting λ DNA (48.5 kbp) using a restriction endonuclease, ApaI, shown in Figure 2a. The successful digestion of λ DNA into two fragments (10 and 38.5 kbp) was confirmed through gel electrophoresis (Figure S3). Nanopore measurements of the unpurified mixture further validated the digestion, exhibiting two distinct populations in the density scatter plot (Figure S4c). After the enzymatic digestion, the 10 kbp fragment was hybridized with the α-Syn probe and purified by using gel extraction (Figure S4d). The α-Syn probe consists of a 12-base sequence complementary to the sticky end of the 10 kbp carrier, along with a 24-mer aptamer that preferentially binds to α-Syn oligomers rather than other α-Syn species.^44^ The 12-base linkage sequence exhibits a high GC content of 83.3%, resulting in a melting temperature, *T*_m_ = 55.5 °C and dissociation constant of 5.22 × 10^–14^ M. These characteristics ensure the sufficient stability of aptamer-carrier complexes for nanopore measurements performed at room temperature.

Compared to λ DNA carriers, using shorter 10 kbp carriers^41^ reduces the probability of DNA folding (Figure S4b). As shown in Figure S4a, long DNA fragments exhibit random folding, which may result in false positives. Typical events of protein-bound carriers are shown in Figure 1c. The DNA carrier, a long linear chain with evenly distributed negative charges, displaces ions in the nanopore sensing region during translocation, resulting in a well-defined blockade signal, as shown in Figure 2c(ii). In contrast, α-Syn possesses nonlinear structures with a slight negative net charge on the surface (pH 8.0).^45^ In addition, α-Syn oligomers, formed from misfolded monomers, vary in size and shape, leading to transient subpeaks in the nanopore readouts that correlate with the extent of α-Syn aggregation (Figure 1b and c). The diameter of the nanometer-sized α-Syn oligomers is much shorter than the length of the dsDNA carrier, causing shorter dwell times for proteins within the nanopore compared to DNA. Instead, the cross-sectional area of the α-Syn oligomers is larger than that of dsDNA, resulting in greater ion exclusion and, consequently, higher current blockade during protein translocation.^46^ Therefore, the binding of α-Syn oligomers can be confirmed by the presence of sharp subpeaks at the ends, and the amplitude of these subpeaks (Δ*I*) can provide insights into the degree of aggregation for individual α-Syn oligomers.

### Detection of α-Syn Oligomers at Various Aggregation Time Points

There is significant evidence that the conversion of soluble α-Syn into highly ordered, cross-β sheet fibrillar structures undergoes a series of transient, soluble oligomeric intermediates.^2^ Nanopores have a confined space that only allows molecules smaller than its dimension to translocate through the pore, thus offering size selectivity on the analyte molecules. In our case, the pore size was 22 ± 3 nm, enabling the detection of oligomers smaller than 20 nm, that are highly related to the formation of pathogenic amyloids and may be crucial in early stage diagnosis and therapy of PD.^3,47^ In order to study α-Syn at the different aggregation time points, we prepared oligomeric samples by aggregating α-Syn monomers from 0.5 to 6 h. These α-Syn oligomers were characterized using atomic force microscopy (AFM), Figure S6. The α-Syn oligomers were then incubated with the aptamer-modified carriers in a binding buffer (100 mM KCl, 50 mM NaCl, 5 mM MgCl_2_, 10 mM Tris-EDTA pH 8.0) before conducting nanopore measurements in the measuring buffer solution (2 M LiCl, 5 mM MgCl_2_, 10 mM Tris-EDTA, pH 8.0). This procedure was intended to stabilize the G-quadruplex structure of the aptamer on the carrier under K^+^/Na^+^ environment,^48,49^ ensuring effective binding of the protein analytes.

Control experiments of the aptamer-modified carriers are shown in Figure S7. The scatter plot of the translocation events for 10 kbp carriers revealed a well-defined population with the mean dwell time and peak current to be 1.3 ± 0.4 ms and 33.6 ± 8.7 pA (mean ± one standard deviation), respectively, consistent with the results reported on a similar nanopore platform.^50^Figure 2c(ii) illustrates typical events corresponding to DNA carriers translocating in a single-file conformation, whereas folded DNA would give rise to a double current blockade (Figure S7d).^51^ These events caused by partial folding could be eliminated by further subpeak analysis without affecting the determination of protein-binding subpeaks.

Typical current–time traces recorded for carrier bound to different α-Syn oligomers (aggregated for 0.5 2, 4, and 6 h, respectively) are shown in Figure 2c(i). As the aggregation time increased, we could observe an increasing proportion of translocation events with a sharp subpeak at either end. The position of these subpeaks depends on whether the protein or the DNA translocated through the pore first. Protein-binding subpeaks were observed alongside folded carriers in Figure S7f but could be easily differentiated based on their higher amplitude and shorter dwell time. The distribution of the fractional subpeak positions is shown in Figure S8, with the beginning and end of individual events defined as 0 and 1, respectively. Possible translocation scenarios are illustrated in Figure S8a. Control measurements with α-Syn oligomers only, without carriers, were also performed with very few translocation events detectable (Figure S9). Scatter plots of peak current versus dwell time, along with respective histograms for detecting α-Syn oligomers, are shown in Figure 2c(iii). It should be noted that a small fraction of events with a dwell time below 0.3 ms were excluded from further data analysis as these events were attributed to the translocation of unbound α-Syn oligomers or other interferants in the solution. The dwell time distributions with and without α-Syn oligomers did not exhibit significant differences, suggesting that the DNA dominated the carrier transport. In contrast, the peak current distribution indicated that the successful binding of α-Syn oligomers to the carriers resulted in a substantial increase in peak current. More importantly, as the aggregation time extended, this current distribution showed a widening trend with an increasing proportion at high current levels, which suggests an increase in the mean size of the detected α-Syn oligomers. The dashed line in the scatter plots represents the threshold used to distinguish protein-bound carrier signals from the carrier alone. Events below the threshold current correspond to unfolded and folded carriers, whereas event populations above the threshold are associated with the binding of α-Syn oligomers.

### Subpeaks Analysis of Time-Dependent α-Syn Oligomers Bound to Aptamer-Modified Carrier

**Figure 3:**
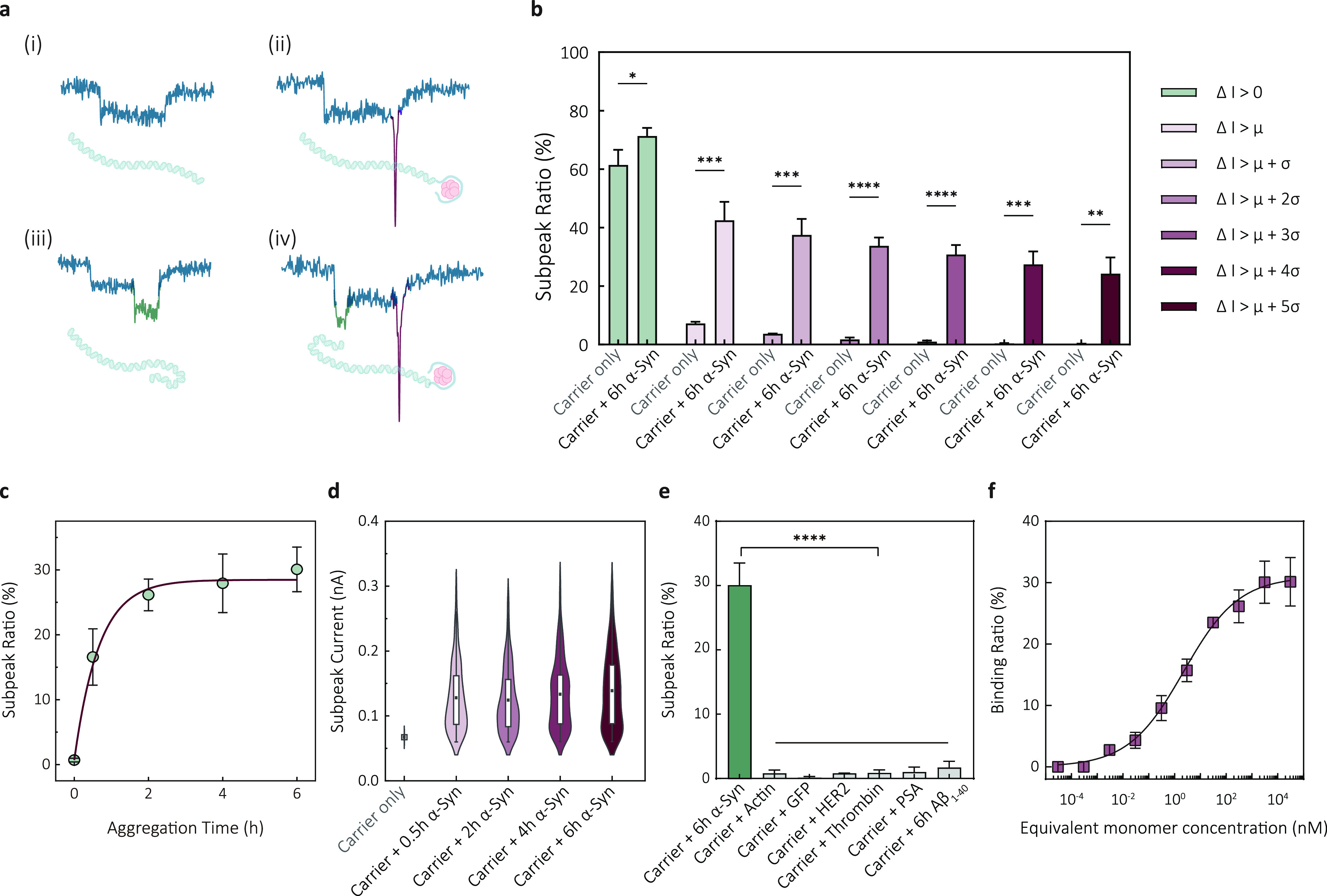
Analysis of subpeaks and evaluation of platform selectivity and sensitivity. (a) Typical signals: (i) unfolded DNA, (ii) DNA–protein binding, (iii) folded DNA, and (iv) DNA–protein binding with partially folded DNA strand. (b) The subpeak ratio between DNA carrier and oligomer-bound DNA carriers was calculated at different thresholds using the mean and standard deviation of the mean. The subpeak ratio is determined by dividing the signals with a subpeak current that exceeds the threshold by the total number of signals. A two-tailed *t* test was used to compare two samples at different thresholds, and significant levels were indicated with asterisks, *p ≤ 0.05; **p ≤ 0.01; ***p ≤ 0.001; ****p ≤ 0.0001. It is worth mentioning that a threshold of mean ±3σ was found to effectively exclude DNA folded signals and enable optimal discrimination between DNA carrier and DNA–protein bound signals. (c) The subpeak ratio of DNA carrier bound α-Syn oligomers at different aggregation time points. (d) Violin plot combined with a box plot of subpeak current distribution of carrier, carrier + 0.5 h, carrier + 2 h, carrier + 4 h, and carrier + 6 h aggregated α-Syn oligomers, respectively. The plot includes a black dot to indicate the mean value, a white bar to represent the interquartile range, and a thin gray line to indicate the rest of the distribution. A kernel density estimation is displayed on either side of the gray line. (e) Subpeak ratio analysis of the selectivity of α-Syn aptamer modified DNA carrier against different proteins. (f) Binding curve of 6 h aggregated α-Syn oligomers bound to the DNA carrier (200 pM). The α-Syn oligomer concentration is plotted as an equivalent monomer concentration. Three nanopore measurements (*n* = 3) were performed for each data point, and the standard deviation of the mean was represented using error bars.

To enhance the accuracy of protein detection and avoid false positives, we extracted protein-bound subpeaks from translocation events. The subpeak current (Δ*I*) was defined as the difference between the peak maximum (*I*_max_) and the DNA level (*I*_0_), i.e., . The partial folding of the carriers was identified as the main cause of false positives, in which case the subpeak amplitude is approximately equal to the DNA translocation current level (), as shown in Figure 3a(iii). To set a threshold for subpeak extraction, we first fitted the distribution of peak current for the carriers using a Gaussian function to determine its mean value (μ) and standard deviation (σ), which was calculated to be 34.2 ± 8.4 pA, based on unfolded DNA carrier peak current, Figure S10. Considering DNA carriers with 6 h aggregated α-Syn oligomers as an example, in Figure 3b, without setting a threshold, no significant difference in the subpeak ratio would be observed between the carriers alone and protein-bound carriers. However, as we raised the subpeak threshold from μ to μ ± 3σ, these two data sets exhibited an increasing statistical significance, suggesting the elimination of most false positives at this threshold. With a further increase of the threshold to μ ± 5σ, the significance would decline, and more protein-bound subpeaks would be lost, Figure 3b.

The subpeak ratio of detected α-Syn oligomers was defined as the number of events with a subpeak beyond the threshold divided by the total number of events and was measured as a function of aggregation time, Figure 3c. Compared to the carriers alone, carriers bound to α-Syn oligomers showed a logarithmic increase in the subpeak ratio over the aggregation time, indicative of the formation and growth of detectable α-Syn oligomers. By applying a simplified kinetic model for early nucleation, the observed time constant for α-Syn oligomerization can be determined to be 0.67 h^–1^.^52^ Our results indicated that the concentration of α-Syn oligomers remained relatively steady after prolonged aggregation. This observation is consistent with the role of α-Syn oligomers as transient intermediates in the amyloid pathway, where they can further aggregate to amyloid fibrils or degrade back into monomers. While the concentration of α-Syn oligomers may not change much, the concealed size heterogeneity can be revealed by the distribution of subpeak current shown in Figure 3d. We could observe an increase in the mean subpeak current over time for α-Syn oligomers, specifically with values of 60.8 ± 5.6 pA (0.5h aggregation), 61.1 ± 5.5 pA (2h aggregation), 69.9 ± 6.2 pA (4h aggregation), and 79.9 ± 6.7 pA (6h aggregation). The upward trend in the mean subpeak current indicates a progressive growth in the size of the α-Syn oligomers over time, suggesting an accumulation of larger oligomeric species. Apart from the mean value, the distribution is more informative, as it can reflect the size distribution of bound α-Syn oligomers. The widening distribution implies an increasing proportion of oligomers with large molecular weight over time, suggesting a transition of overall α-Syn toward fibrillar amyloids.

Subpeak dwell time analysis for different samples is shown in Figure S11c. Signals with subpeak current exceeding the threshold of μ ± 3σ pA were selected to analyze their subpeak dwell time. The mean dwell time of protein-bound subpeaks was significantly lower than that caused by DNA folding, as observed in Figure S11c. This decrease in dwell time was attributed to the fast translocation speed of proteins.^53^Figure S11b illustrates that protein-bound subpeaks were mostly populated at a short dwell time range (<0.1 ms) with a higher amplitude. Compared to the translocation of pure α-Syn proteins, where most events were undetected, DNA carriers could somewhat slow the protein translocation and increase the ratio of detectable protein-binding events.

### Selectivity and Sensitivity of the Detection Platform

We conducted a selectivity experiment to confirm that the presence of subpeaks is specific to the binding of α-Syn oligomers rather than to other common proteins present in the biofluids. To assess selectivity, we compared the subpeak ratio between 6 h aggregated α-Syn oligomers and a protein mixture consisting of actin, GFP, HER2, PSA, thrombin, and amyloid-beta (Aβ_1–40_), as shown in Figure 3e. Aβ oligomers were specifically tested as many amyloid fibrils share a similar cross-β structure,^2^ which may pose a challenge in distinguishing α-Syn oligomers from structurally similar amyloids. In all experiments, the carriers were incubated with the protein at a ratio of 1:100 in the binding buffer for 2 h and then diluted into the final 200 pM carrier concentration and 20 nM protein concentration in the measuring buffer for nanopore translocation measurements. Subpeaks exceeding  were classified as protein-bound signals. We observed a low subpeak ratio for the protein mixture comparable to the carrier only. By contrast, incubation with α-Syn oligomers led to an apparent increase in the subpeak ratio. We observed nearly 10-fold enhancement, indicating that the α-Syn oligomer aptamer and the detection method exhibited excellent specificity.

Quantifying the concentrations of α-Syn oligomers and fibrils may be crucial for clinical applications, such as potential early disease detection, tracking disease progression, and assessing treatment efficacy. Oligomers, in particular, are present at very low concentrations in biofluids and can initiate a cascade of pathological events leading to cell death.^10^ To evaluate the binding assay, we measured 200 pM carriers with 6 h aggregated α-Syn oligomers with concentrations ranging from 20 fM to 20 μM (equivalent monomer concentration), as shown in Figure 3f. The binding ratio increased with increasing α-Syn oligomer concentration, following the expected sigmoid curve shape. By fitting the binding curve with the Hill–Langmuir equation, we determined *K*_d_ to be 1.6 nM. Notably, this value is much lower than that obtained for the aptamer sequence alone through blotting assays (63 nM).^44^ The improved binding affinity observed in our approach can be attributed to the high sensitivity of nanopore sensors, which offer single-molecule resolution and the ability to detect transient oligomeric intermediates. The limit of detection (LOD), defined as 3σ above the background noise, was estimated to be approximately 2.2 pM. One advantage of single-molecule detection is that because the ratio of bound vs unbound oligomers is established by measuring one molecule at a time, it is possible to detect concentrations lower than the *K*_d_ and close to the LOD, if sufficient data are collected. In addition, the LOD can be improved further by changing the concentration of DNA carriers. It is worth noting, however, that the binding ratio reaches saturation and plateaus at a maximum binding ratio of approximately 30% after reaching micromolar concentrations. This saturation can be attributed to the weak binding of α-Syn oligomers to the aptamer at high ionic strength. The high ionic strength can shield charge interactions and lead to a lower affinity, particularly when the aptamer concentration is low, as in this work (200 pM). Since we focused on the early stages of α-Syn aggregation, with a maximum aggregation time of 6 h, most α-Syn species in the sample are either monomeric or small oligomers. As the size of the oligomers bound to the DNA carrier decreases, they become more difficult to detect. This challenge arises from the reduced SNR due to their smaller size and the resolution constraints of nanopore detection.^54^

### Single-Molecule Sensing of α-Syn Oligomers Directly from Unprocessed CSF

Human cerebrospinal fluid is a body fluid derived from blood plasma that circulates within the ventricular system of the brain.^55^ Unlike blood plasma, CSF is nearly devoid of proteins, making it a favorable environment for detection with minimal background interference. Secreted forms of α-Syn have been observed in both mouse and human brains, with significant levels found in the interstitial fluid in cases of both PD and non-PD individuals.^56^ However, the composition of α-Syn oligomers formed in the CSF may vary under different conditions, such as salt concentration, pH, and temperature, which can complicate direct detection from CSF.^57^

**Figure 4:**
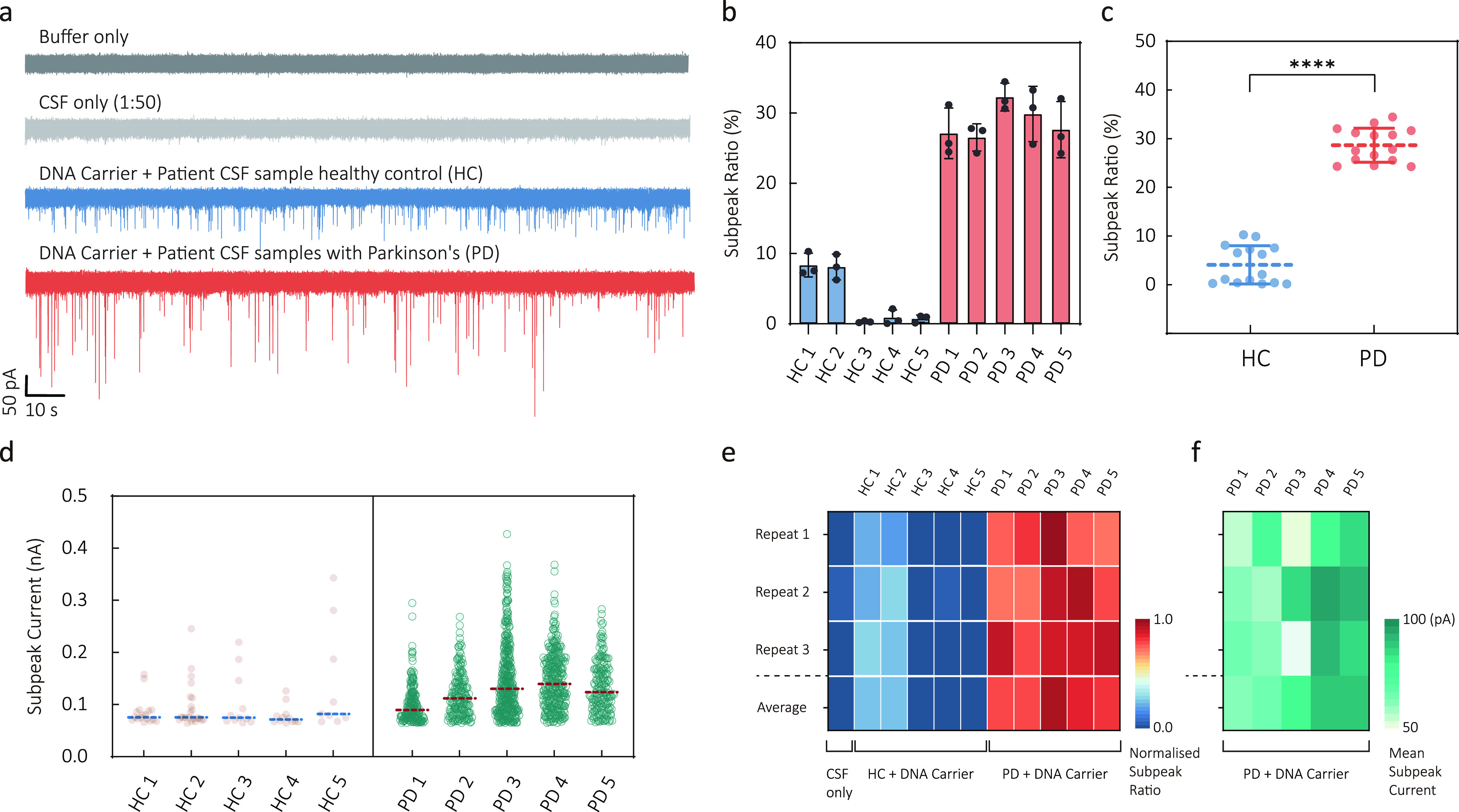
Detection of α-Syn oligomers directly from patient cerebrospinal fluid. (a) Typical current–time traces are shown for buffer only, CSF only, carriers incubated with healthy control CSF sample (HC), and carriers incubated with PD patient CSF sample (PD). (b) Subpeak ratios of the healthy control cohort (*n* = 5) and PD patient cohort (*n* = 5). The data are presented as mean ± σ. (c) Scatter plots show subpeak ratios for all healthy and patient cohorts. Statistical significance was evaluated using a two-tailed *t* test, and the result was *p* < 0.0001. (d) Scatter dot plots of subpeak current for healthy and PD patient cohorts. The dashed lines represent the mean values. (e) Heatmap of the normalized subpeak ratio of CSF sample, carriers incubated with healthy controlled CSF, and carriers incubated with PD patient CSF. (f) Heatmap of the normalized mean subpeak current of carriers incubated with PD patient CSF. Each sample was repeated in three nanopores, and the average represents the mean value of three repeats.

The preliminary nanopore results obtained from purified α-Syn oligomers offered the possibility of detecting α-Syn oligomers directly in the patient samples. To validate feasibility, we conducted double-blinded experiments using CSF samples from two cohorts: a patient cohort of individuals clinically diagnosed with PD (*n* = 5, PD1-PD5) and a healthy control cohort (*n* = 5, HC1-HC5). The two cohorts (each comprising three female and two male patients) were age-matched. To mitigate variations caused by the complex components in CSF, we initially investigated the impact of CSF concentrations on ionic current measurements. We observed significant fluctuations in the ionic current at high CSF concentrations (1:2, CSF in buffer, v/v), as shown in Figure S12a. These fluctuations were attributed to nanopore clogging, resulting from the concentrated background molecules. The high background noise level could distort the current baseline and overshadow the signals from carriers, thereby introducing bias into the detection results. To address this issue, we determined an optimal CSF dilution (1:50, v/v) that exhibited a stable baseline and a low noise level comparable to that of pure buffer (Figure 4a). This dilution condition was subsequently employed in further experiments, where 200 pM DNA carriers were added and incubated.

As shown in Figure 4a, introducing carriers into the diluted CSF samples from healthy controls resulted in typical carrier translocation events. For the PD patient samples, we could observe an apparent increase in the number of events with a subpeak in the current–time traces. Those signals closely resemble the results observed by using carriers to detect purified α-Syn oligomers. Moreover, an analysis of signals from these patients with a clinical diagnosis of PD reveals a distinct signal pattern characterized by relatively higher subpeak current and longer subpeak dwell time, as shown in Figure S12b(iii). This finding suggests the presence of amyloid fibrils formed through aggregation in the patients' CSF.

We performed subpeak analysis on individual samples, as shown in Figure 4b–e. In terms of the subpeak ratio, PD patients exhibit an overall higher fraction than the healthy control group in Figure 4b, aligning with the expectation that PD patients have an increased concentration of α-Syn oligomers in their CSF. HC1 and HC2 show relatively higher subpeak ratios than other controls but still lower than the confirmed PD patients, suggesting that the detection can reveal intrinsic variability across different samples. The subpeak ratio of the two cohorts was analyzed using a two-tailed *t* test (Figure 4c). The *p*-value analysis in Figure 4c indicates that our method can unveil significant differences between healthy and patient samples, indicating a promising potential application in supporting clinical diagnosis. Given the low concentrations of α-syn oligomers in human CSF, a standard addition of exogenous α-Syn oligomers was performed using the CSF sample from HC1 to validate the feasibility in clinical diagnostics, as shown in Figure S13. By fitting the linear region at low concentrations, the background concentration of the α-Syn oligomers in this CSF sample was found to be 0.8 pM (equivalent monomer concentration).

In addition to the subpeak ratio analysis, the distribution of the subpeak current can also reveal the inherent heterogeneity of α-Syn oligomers within individual CSF samples. This analysis elucidates two key factors highly relevant to the aggregation state of α-Syn proteins: the mean subpeak current and the distribution range, Figure 4d. The mean value of the subpeak current reflects the average size of α-Syn oligomers in the CSF, while the range visualizes the size heterogeneity and the aggregation tendency of α-Syn. When the mean subpeak current of the healthy control cohort is compared to that of the PD patient cohort, it is lower in the former and higher in the latter. Furthermore, the subpeak current distribution is much broader for the PD patient cohort, suggesting a larger quantity of α-Syn oligomers with varying sizes.

### Correlation between Nanopore Readouts and Clinical Diagnosis

We further explored the correlation between the nanopore analysis results and disease severity, measured by the duration of the disease in years since diagnosis. To establish the correlation, we evaluated four different methods using the normalized values of the following nanopore sensing variables in (i) mean subpeak ratio, (ii) subpeak current range (maximum-minimum), (iii) subpeak current standard deviation, and (iv) mean subpeak current. The correlation analysis used Pearson and Spearman coefficients, Figure S14. We found that all nanopore readouts positively correlated with the PD duration based on clinical diagnosis. A progressive increase in Pearson’s r value from (i) to (iv) indicated a stronger positive correlation. This suggests that the mean subpeak current exhibits the most substantial positive correlation with the PD duration. The mean subpeak current and standard deviation of subpeak current demonstrated higher correlations, with Spearman’s correlation coefficient R being greater than 0.8. Both correlation coefficients suggest that the distribution of subpeak current obtained from the nanopore analysis may indicate the state of α-Syn oligomers and provide valuable insights into monitoring the progression of PD.

We generated a heatmap of the normalized subpeak ratio for healthy and patient cohorts in Figure 4e−f. that indicated a clear differentiation between the healthy controls and PD patients. A color map of the mean subpeak current further reveals the single-molecule heterogeneity among individual PD patients. These results were obtained by repeating the experiments with at least three nanopores.

## Conclusion

The nanopore sensing technology offers numerous potential advantages for detecting protein-bound carriers with enhanced sensitivity and selectivity, high throughput, and the ability to achieve single-molecule resolution. Compared with conventional techniques such as fluorescence microscopy and immunoassays, our platform enables accurate and selective identification of α-Syn oligomers without the need for α-Syn monomer fluorescent labeling, which could alter and disrupt the conformation and kinetics of oligomer aggregation. Furthermore, using aptamers in our technique allows for direct discrimination between PD patients and control cohorts using clinical samples, overcoming the limitations associated with time-consuming and costly sample preparation, purification, and bulk measurements that typically require larger sample volumes. Remarkably, the platform can achieve reliable results using just 1.5 μL of an unprocessed CSF sample, dramatically reducing the volume of the sample collected via lumbar puncture.

The carrier design is fully customizable and can facilitate the identification of potential biomarkers and proteins implicated in neurodegenerative disorders and diseases caused by protein misfolding and aggregation, including cancers associated with p53 aggregation.^58^ This technology can be adapted to target different protein biomarkers in various diseases by simply replacing the aptamer sequences. In the case of neurodegenerative disorders like Parkinson’s disease, Alzheimer’s disease (AD), and Lewy Body Dementia (LBD), misdiagnosis is a significant concern due to the overlap in symptoms despite distinct mechanisms and pathogenesis. In addition, by incorporating multiplexed detection in the nanopore system, where different DNA carriers can selectively detect specific biomarkers of interest (e.g., α-Syn oligomers and tau proteins), it may further become feasible to distinguish between different biomarkers and improve diagnostic accuracy.^41^

## Methods

### Preparation of 10 kbp DNA Carrier

λ-DNA (48.5 kbp, 500 μg/mL, New England Biolabs) was designed for digestion using ApaI restriction enzyme (50,000 U/ml, New England Biolabs) at a specific site, resulting in two fragments: one comprising 10 kbp and the other 38.5 kbp. 10 kbp DNA fragment was selected as a DNA carrier. To prepare the digested solution, 25 μL of stock λ-DNA, 10 μL of 10x CutSmart buffer, 5 μL of ApaI, and 60 μL of deionized water, resulting in a final volume of 100 μL. This solution was incubated at 25 °C for 30 min to facilitate digestion, followed by heating to 65 °C to inactivate the enzyme. The 10 kbp DNA carriers, featuring 12-base overhangs, were subsequently hybridized with a complementary sequence-extended α-Syn aptamer at a ratio of 1:100. The aptamer sequence was GGTGCGGCGGGACTAGTGGGTGTG (T-SO530).^44^ Hybridization took place in a buffer solution containing 100 mM KCl, 5 mM MgCl_2_, and 10 mM Tris-EDTA, utilizing a PCR annealing device (TC-3000, TECHNE). The hybridization process involved an initial heating step to 75 °C for 5 min, followed by a gradual cooling process at 1 °C/min until a final temperature of 15 °C was reached. The sample was then loaded on 0.6% (wt %) agarose gel running at 120 V for 180 min to separate two fragments and extract α-Syn aptamer modified 10 kbp DNA carrier using Monarch DNA Gel Extraction Kit (New England Biolabs). The concentration of the aptamer-modified DNA carrier was determined with a Nanodrop 2000c instrument (Thermo Fisher Scientific), measuring the UV–vis absorbance at 260 nm.

### Preparation of α-Syn Oligomers

α-Syn monomers were expressed using recombinant expression in *Escherichia coli*. The concentration was estimated from the absorbance at 275 nm by using a molar extinction coefficient of 5600M^–1^·cm^–1^. 800 μL of α-Syn monomers (70 μM, 25 mM Tris, 100 mM NaCl, pH = 7.4) were prepared to ultracentrifugation for 1 h at 90,000 rpm and 4 °C using the Thermo Scientific Sorvall MTX 150 Micro-Ultracentrifuge. The top 600 μL of the solution was collected, while the remaining volume was discarded. Next, 400 μL of the centrifuged sample was mixed with 0.01% NaN_3_ to prevent bacterial growth during aggregation. The mixture was then used for aggregation by shaking in the Digital Heating Shaker Drybath (ThermoFisher) at a constant shaking speed of 200 rpm and a temperature of 37 °C. At specific time points (0.5, 2, 4, and 6 h), aliquots of 20 μL were removed from the incubated sample and stored in a refrigerator at 4 °C prior to use as previously reported.^13^ The ThT binding assays shown in Figure S5 verify that the α-Syn aggregates did not further grow significantly after being stored at 4 °C. It is important to note that the stored samples should be used within 1 week to ensure their accuracy and consistency.

### Preparation of DNA–Protein Complexes: Hybridization of the Aptamer to DNA Carrier and α-Syn Oligomer Binding Experiments

The concentration of α-Syn oligomers, equivalent to monomers, was diluted at various concentrations in phosphate-buffered saline (PBS; 137 mM NaCl, 2.7 mM KCl, 8.1 mM Na_2_HPO_4_, 1.4 mM KH_2_PO_4_, pH = 7.3). The α-Syn aptamer modified DNA carriers (2 nM) were then incubated with different α-Syn oligomers collected at various time points for 2 h under constant shaking at 200 rpm to allow complete reaction. The prepared proteins bound carriers were further diluted using 2 M LiCl solution to achieve a final carrier concentration of 200 pM. For the clinical sample, α-Syn aptamer modified DNA carriers (2 nM) were incubated with 1.5 μL of unprocessed CSF samples for 2 h at a constant shake of 200 rpm. Then the samples were diluted using 75 μL of 2 M LiCl measuring buffer to achieve a final carrier concentration of 200 pM. The 10 CSF samples were provided and selected by the Parkinson’s UK Brain Bank to have similar ages and random sex. Specifically, 5 samples are from PD patients who passed away due to this disease, and the other 5 are from healthy elderly individuals without clinical PD symptoms. More detailed information about these samples can be found in Table S1 in the Supporting Information.

### Fabrication of Nanopipettes

All the nanopipettes were fabricated by pulling quartz capillaries (GQF100-50-7.5, World Precision Instruments, UK) using a laser-based micropipette puller (Sutter Instrument, P-2000, USA) as per protocol reported previously by our group. Prior to pulling, the capillaries (inner diameter: 0.5 mm, outer diameter: 1.0 mm, length: 7.5 cm) were cleaned thoroughly for approximately 30 min using a plasma cleaner (Harrick Plasma) to remove any organic residues or contaminants on the quartz surface. A capillary was then set up on the capillary holder of the puller and pulled using an optimized two-line protocol: (1) HEAT: 775; FIL: 4; VEL: 30; DEL: 170; PUL: 80, (2) HEAT: 825; FIL: 3; VEL: 20; DEL: 145; PUL: 180. Nanopipettes generated using this protocol have pore sizes averaged at 22 ± 3 nm (n = 5), according to the SEM characterization and conductance calculation, as shown in Figures S1 and S2. The pulling protocols are specific to the instrument and are very sensitive to factors such as the laser pathway of the puller, the humidity, and room temperature.

### Nanopore Translocation Experiments

Nanopore translocation experiments were performed from the bath to the inner nanopipette reservoir unless otherwise specified. In this work, ∼ 1.5 μL of 2 M LiCl solution in electrolyte solution was backfilled into the nanopipet using a MicroFil syringe (Microfills 34-gauge 67 mm, WPI) and an Ag/AgCl electrode was inserted to serve as the patch electrode. The nanopipet was then set up on a clamp and immersed in the bath. The sample was placed in the bath, and another electrode was placed, acting as the QRCE electrode. Unless otherwise stated, all samples were diluted in a 2 M LiCl solution and detected at +300 mV at room temperature. Measurements were done with an Axopatch 200B patch clamp amplifier (Molecular Devices, USA) and digitized using Digidata 1440A. The signals were recorded at 250 kHz sampling rate and 100 kHz low-pass Bessel filter, then refiltered at 10 kHz.

### Data Analysis

The analysis of all ionic current recordings^46^ was conducted using a custom-written MATLAB App (The Nanopore App v.7.17). The procedure involved in the processing and analyzing steps can be clarified as the following steps: (1) Loading current–time trace. (2) Refiltering the trace with 10 kHz low-pass filter. (3) Tracking the baseline of the trace. (4) Defining open pore current and threshold through Poisson probability distribution function. (5) Identifying peaks when signals exceeded the predefined thresholds. (6) Analyzing and exporting peak statistics by extracting peak-related information, including dwell time, peak current, and charge. (7) Isolating events that meet established requirements of thresholds, then exporting the subpeak information, including subpeak current, subpeak dwell time, and fractional position. Additional information is available in the Supporting Information.

### Ethics Statement.

The cerebrospinal fluid samples were supplied by the Parkinson’s UK Brain Bank at Imperial College London, an esteemed charitable organization registered in both England and Wales (258197) and Scotland (SCO37554). All samples and associated clinical information are rendered nonidentifiable to researchers upon release. All participants involved in this study had conscientiously given their written informed consent for the donation of brain tissue. The tissue bank was ethically approved by the Research Ethics Committee for Wales (ref 18/WA/0238). Researchers ensure the storage, use, and disposal of the samples in accordance with the HTA Codes of Practice, the terms of the ethical approval, and any other conditions required by the Bank. This work represents a collaborative effort that would not have been feasible without the invaluable resources and dedication of the Parkinson’s UK Brain Bank. We sincerely appreciate the individuals and their families who selflessly donated brain tissue, enabling vital research progress in understanding neurological disorders.

## Supporting Information Available

Electrical conductance characterization of nanopores; electron microscopy characterization of nanopores; gel electrophoresis of ApaI-digested λ DNA; nanopore characterization of λ DNA and digested 10 kbp DNA; ThT fluorescence assay of α-Syn aggregation; AFM characterization of α-Syn oligomers; comparison of signals from 10 kbp carriers, α-Syn aptamer modified carriers, and α-Syn bound carriers; the fractional position for the subpeaks; nanopore detection of pure 6 h α-Syn oligomers; thresholding of protein-bound subpeaks; subpeak analysis of protein-bound events; clinical characteristics of Parkinson’s disease and healthy control participants; nanopore detection of α-Syn oligomers in real CSF samples; standard addition assay of α-Syn oligomers in real CSF samples; correlation between nanopore readouts and duration of Parkinson’s disease; data processing and analysis (PDF)
