# Supplementary material for: Single-Molecule Detection of α-Synuclein Oligomers in Parkinson’s Disease Patients Using Nanopores
Source: ACS Nano. 2023 Nov 10;17(22):22999–3009. doi: 10.1021/acsnano.3c08456 (PMC10690843; doi:10.1021/acsnano.3c08456)
Supplement: Supplementary file 1 — nn3c08456_si_001.pdf [file nn3c08456_si_001.pdf]

Supporting Information

Single-Molecule Detection of α-Synuclein Oligomers in Parkinson's Disease Patients Using Nanopores

Yaxian Liu [a], *Xiaoyi Wang* [a], *Giulia Campolo* [a], *Xiangyu Teng* [a], *Liming Ying* [b], *Joshua B. Edel* [a,*], *Aleksandar P. Ivanov* [a,*]

[a] Department of Chemistry, Imperial College London, Molecular Sciences Research Hub, London, W12 0BZ, United Kingdom.

[b] National Heart and Lung Institute, Imperial College London, Molecular Sciences Research Hub, London, W12 0BZ, United Kingdom.

Corresponding Author Email: joshua.edel@imperial.ac.uk; alex.ivanov@imperial.ac.uk

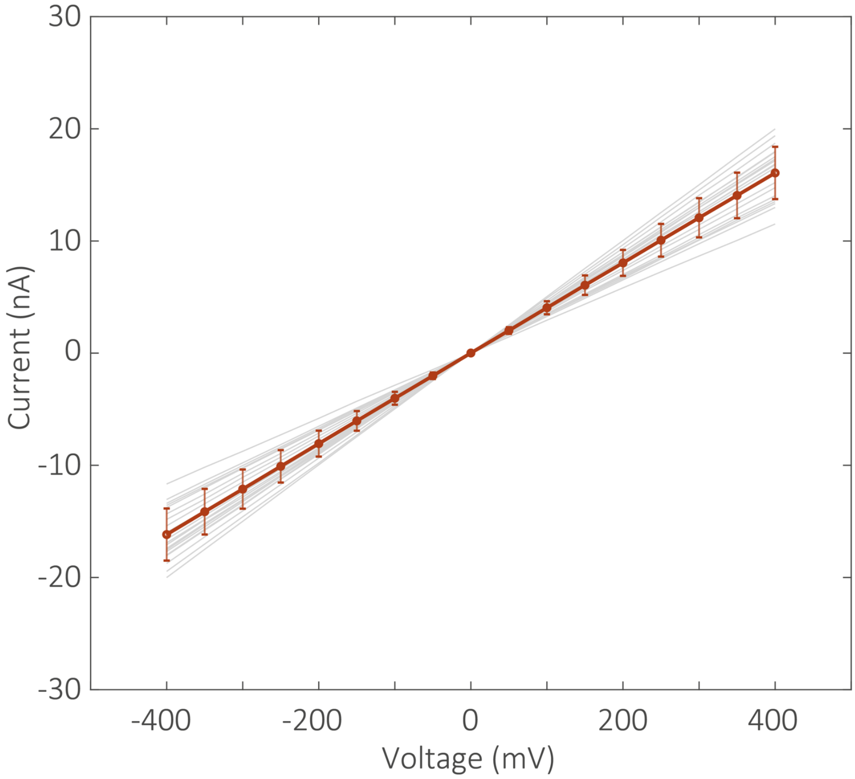

Figure S1. Electrical conductance characterization of nanopores. Current-voltage (I - V) measurements of 20 different quartz nanopipettes were performed in the measuring buffer containing 2 M LiCl, 5 mM $MgCl_2$, and 10 mM Tris-EDTA. The mean conductance of these nanopipettes was determined to be 40.3 ± 5.7 nS (n = 20) by calculating the range from - 400 mV to + 400 mV in the I-V plots. The red points and the error bars correspond to the mean value of 20 measurements and one standard deviation, respectively.

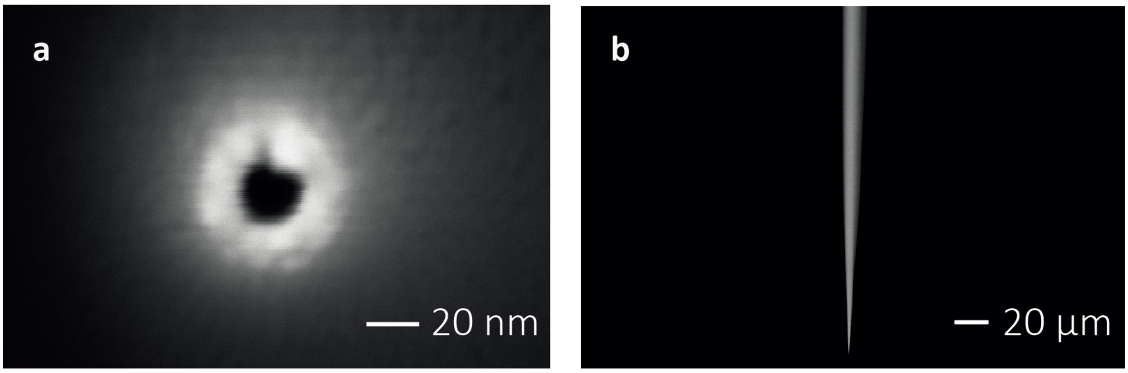

Figure S2. Electron microscopy characterization of nanopores. Representative SEM images of **(a)** top-view and **(b)** side-view of a nanopipette showing its dimension and geometry. The average diameter measured by SEM is 22 ± 3 nm (n = 5; the error represents one standard deviation).

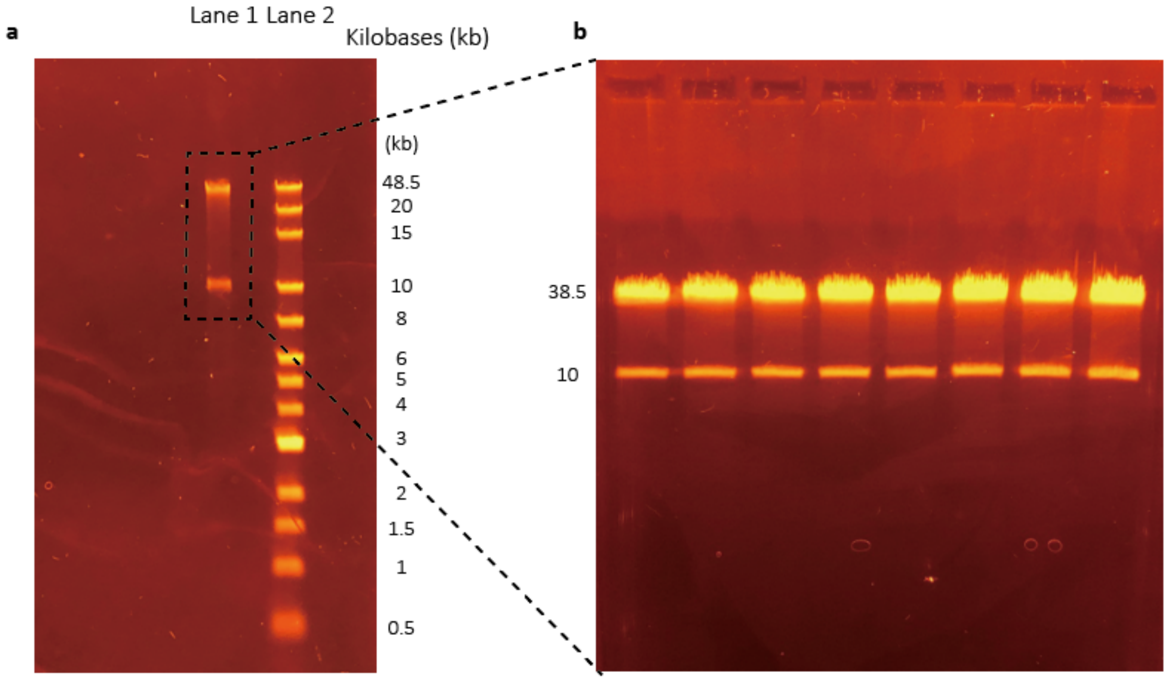

Figure S3. Gel electrophoresis of ApaI-digested λ DNA. 10 kbp DNA carrier was digested from λ DNA using ApaI restriction enzyme, which recognizes the specific 5'…GGGCC/C…3' sequence. Gel electrophoresis was then performed to characterize and purify the 10 kbp fragments from the mixture. **(a)** Gel image of the ApaI-digested λ DNA fragments (Lane 1) alongside a 1 kbp extended DNA ladder (Lane 2) displaying the expected two fragments (10 kbp and 38.5 kbp) after enzymatic digestion. **(b)** A scale-up gel electrophoresis was used to prepare 10 kbp DNA carriers. The bands corresponding to the 10 kbp fragments were cut from the gel and extracted using Monarch® DNA Gel Extraction Kit.

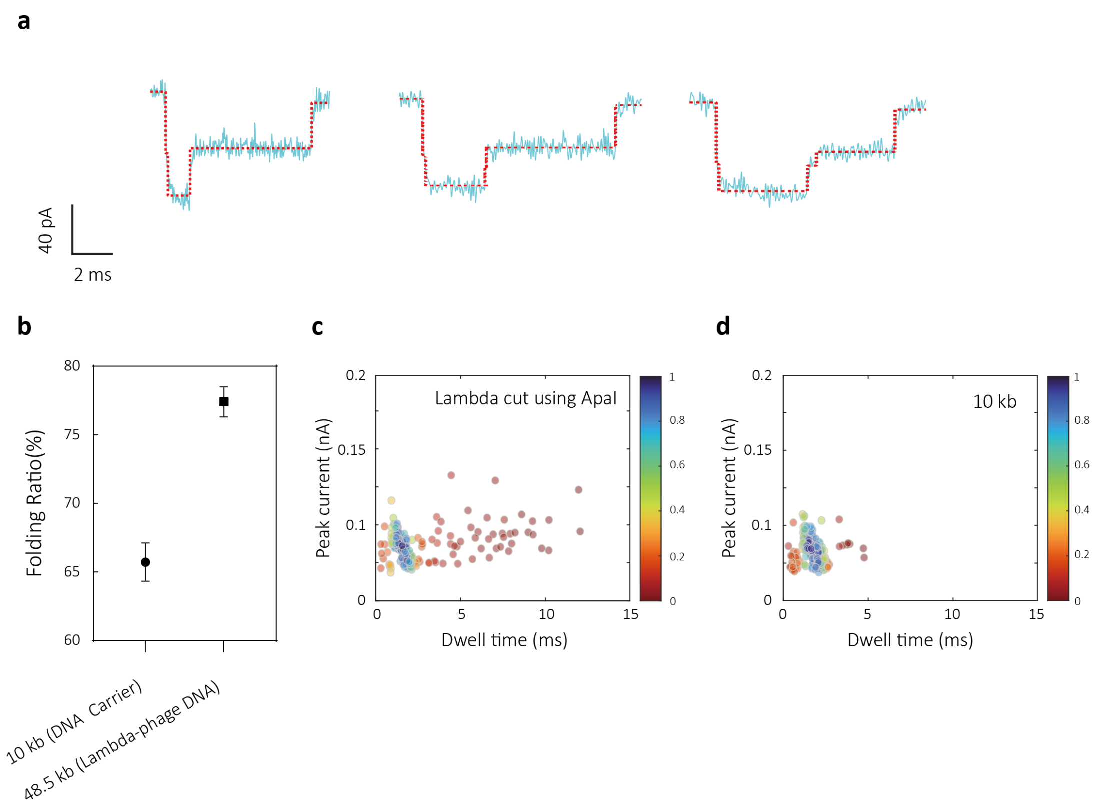

Figure S4. Nanopore characterization of λ DNA and digested 10 kbp DNA. (a) Representative translocation events of folded λ DNA. **(b)** Comparison of the folding ratio between the 10 kbp DNA carrier and the 48.5 kbp λ DNA. The λ DNA with a longer length exhibits a significantly higher folding ratio. **(c)** Density scatter plots of peak current versus dwell time for the unpurified mixture of ApaI digests. Two populations corresponding to 10 kbp and 38.5 kbp fragments were observed in the plots, with differences in dwell time measured as 1.8 ± 0.7 ms and 6.3 ± 5.4 ms, respectively. **(d)** Density scatter plots of peak current versus dwell time for the purified 10 kbp DNA carriers. One population was observed in the plots, with the dwell time measured as 1.5 ± 0.5 ms (mean ± one standard deviation).

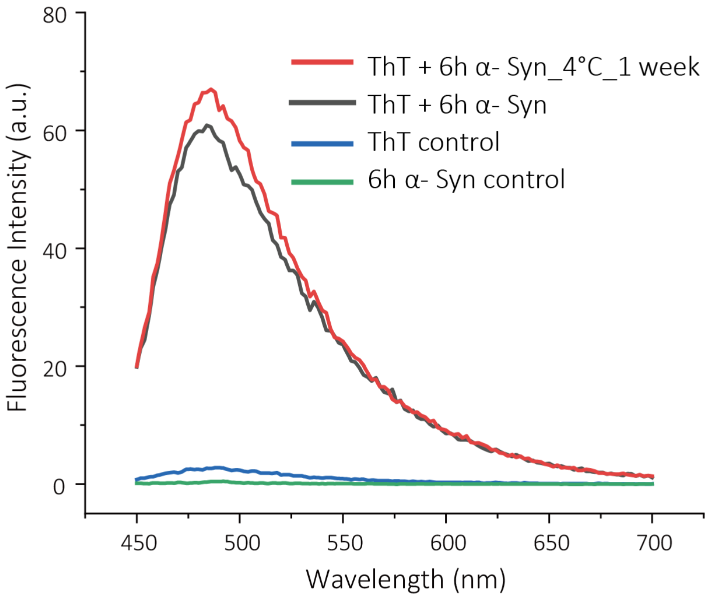

Figure S5. ThT fluorescence assay of α-Syn aggregation. α-Syn samples after 6 h aggregation (black) and samples after 6 h aggregation followed by one-week fridge storage (red) were measured using ThT binding assay. The fluorescence spectrum was recorded under the excitation wavelength at 412 nm. In the fluorescence assays, 20 μM of α-Syn samples were incubated with 30 μM ThT in PBS for 30 minutes before measurements. Control experiments were performed with 20 μM of 6 h α-Syn samples without ThT (green) and 30 μM of ThT only (blue).

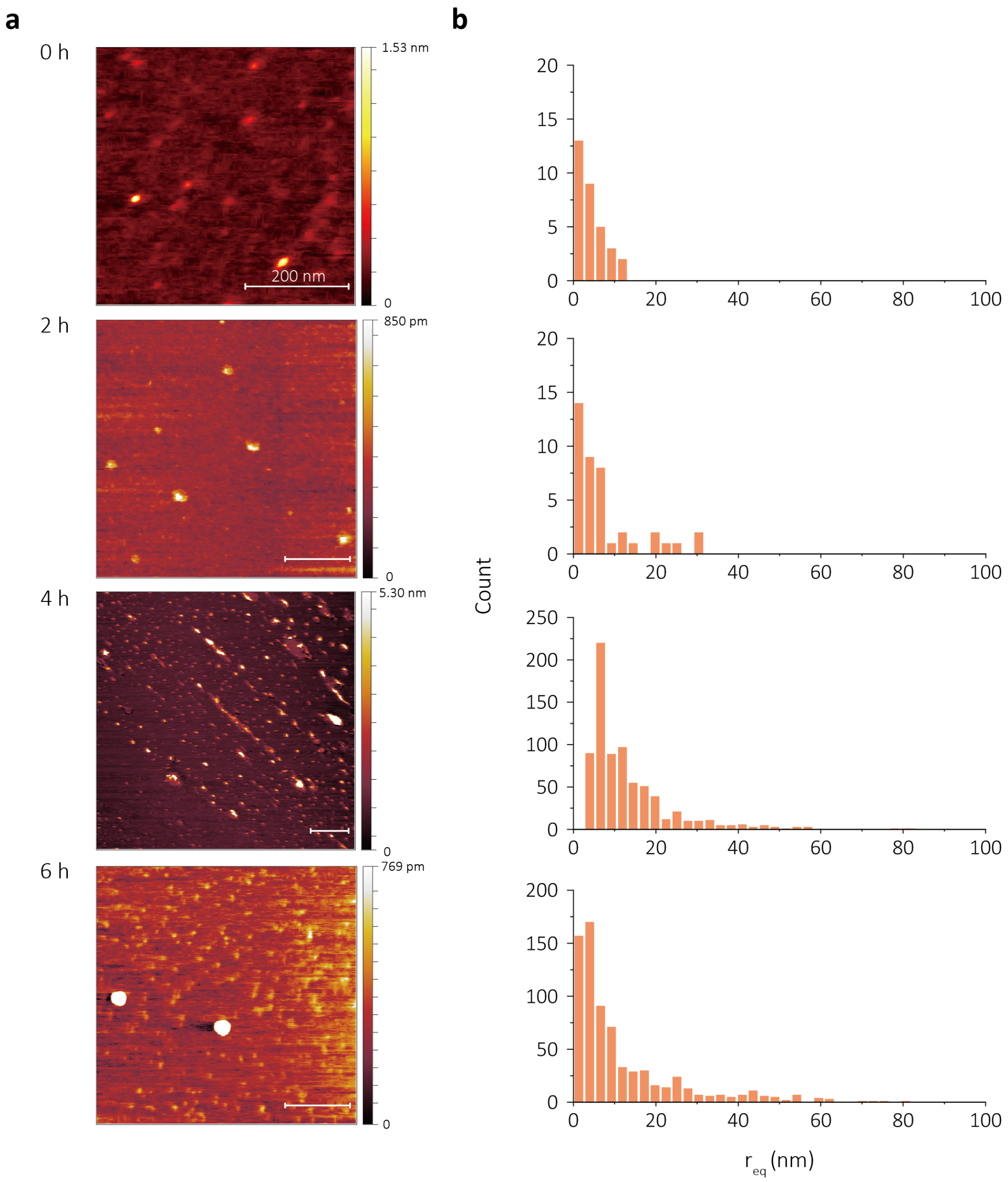

Figure S6. AFM characterization of α-Syn oligomers. (a) AFM scanning images of α-Syn oligomers (0, 2, 4, and 6h). **(b)** Histograms of the equivalent disk radius (r_{eq}) for the particles observed in panel a. α-Syn oligomers were aggregated in the same condition and collected at different time points before the AFM imaging. During this 6h aggregation process, the size of oligomers and aggregates formed gradually increased as aggregation time increased. The observed increasing trend of r_{eq} from 0 h to 6 h, in terms of mean value and distribution range, indicates a progressive aggregation of the α-Syn, which can be attributed to the nucleation of monomeric species into small oligomers. The initial oligomers, detected at 0 h, were relatively smaller in size, but as the incubation progressed, the oligomers exhibited an increase in size and a broader size distribution. These results agreed with the observations in the nanopore analysis. The AFM imaging was performed using the Asylum MFP-3D microscope in the tapping mode. Nanosensors PPP-FMR tips (res ~75 kHz, nom. tip radius 7 nm, nom. spring constant 2 N/m) were used and tuned to a target tapping amplitude of 1 V. The scan parameters were 90 μm^2 in scan size, 512 points per line, and a scan rate of 0.5 Hz. 12 μL of the α-Syn solution was added onto the mica sheets for 5 minutes, then dried with a nitrogen gun or compressed air.

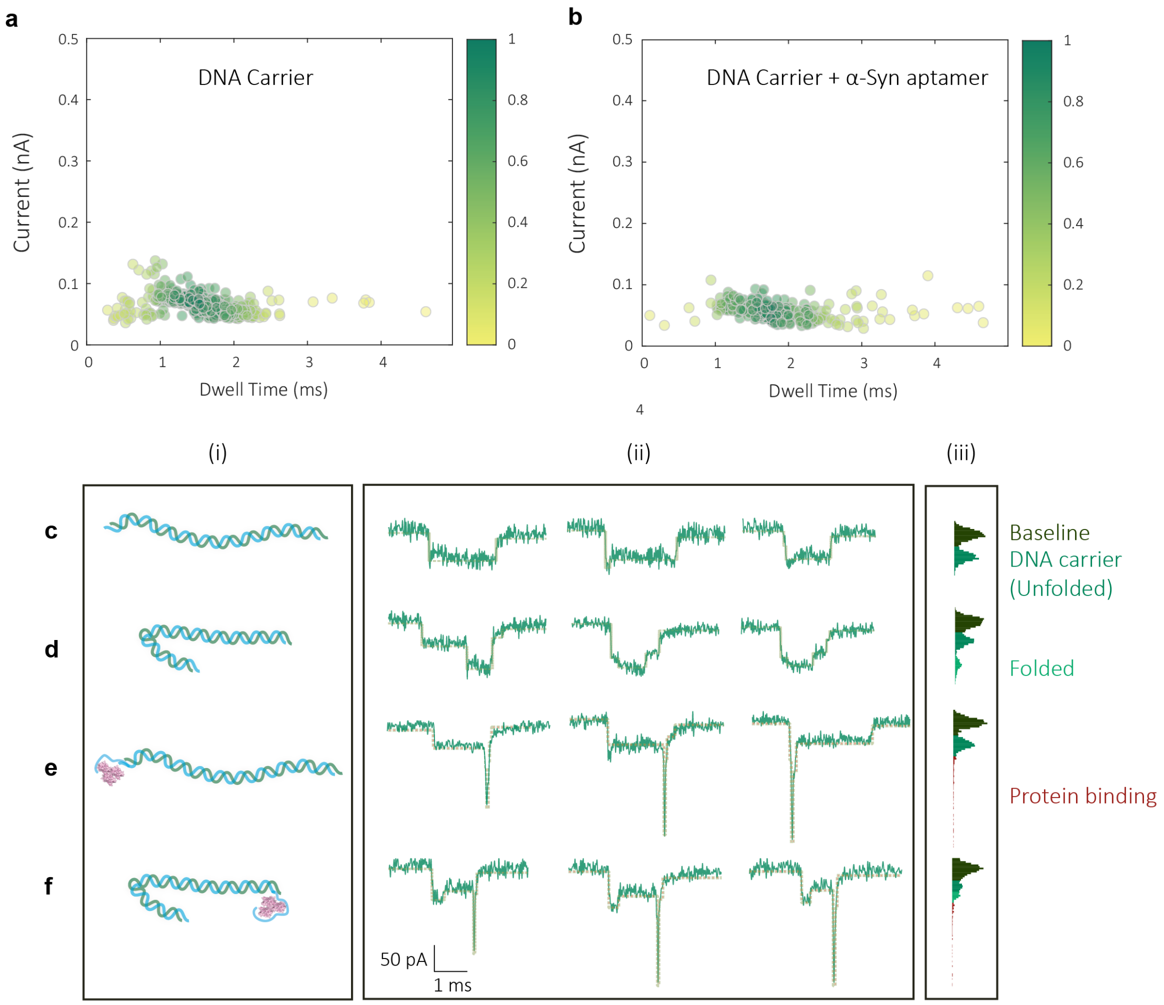

Figure S7. Comparison of signals from 10 kbp carriers, α-Syn aptamer modified carriers, and α-Syn bound carriers. Density scatter plots comparing peak current against dwell time are presented for (**a**) unmodified DNA carriers and (**b**) α-Syn aptamer-modified carriers. The addition of the α-Syn aptamer, with a much shorter sequence length than the 10 kbp carrier, is negligible during the nanopore measurements. High similarities were exhibited in the populations between the unmodified carrier and the aptamer-modified carrier. Typical events of **(c)** linear unfolded carriers, **(d)** folded carriers, **(e)** protein bound with unfolded carriers, and **(f)** protein bound with partially folded carriers. These are illustrated with corresponding schematic representations. The panels are arranged from left to right, with each panel showing three components: (i) schematic, (ii) three characteristic signals, and (iii) a normalized all-points histogram depicting the current-time distribution from the three signals. The histogram showcasing the open pore current is in dark green. The unfolded carrier current distribution is represented in a green histogram, while the lighter green histogram corresponds to the folded carrier. Lastly, the red histograms characterize the events where the protein is bound.

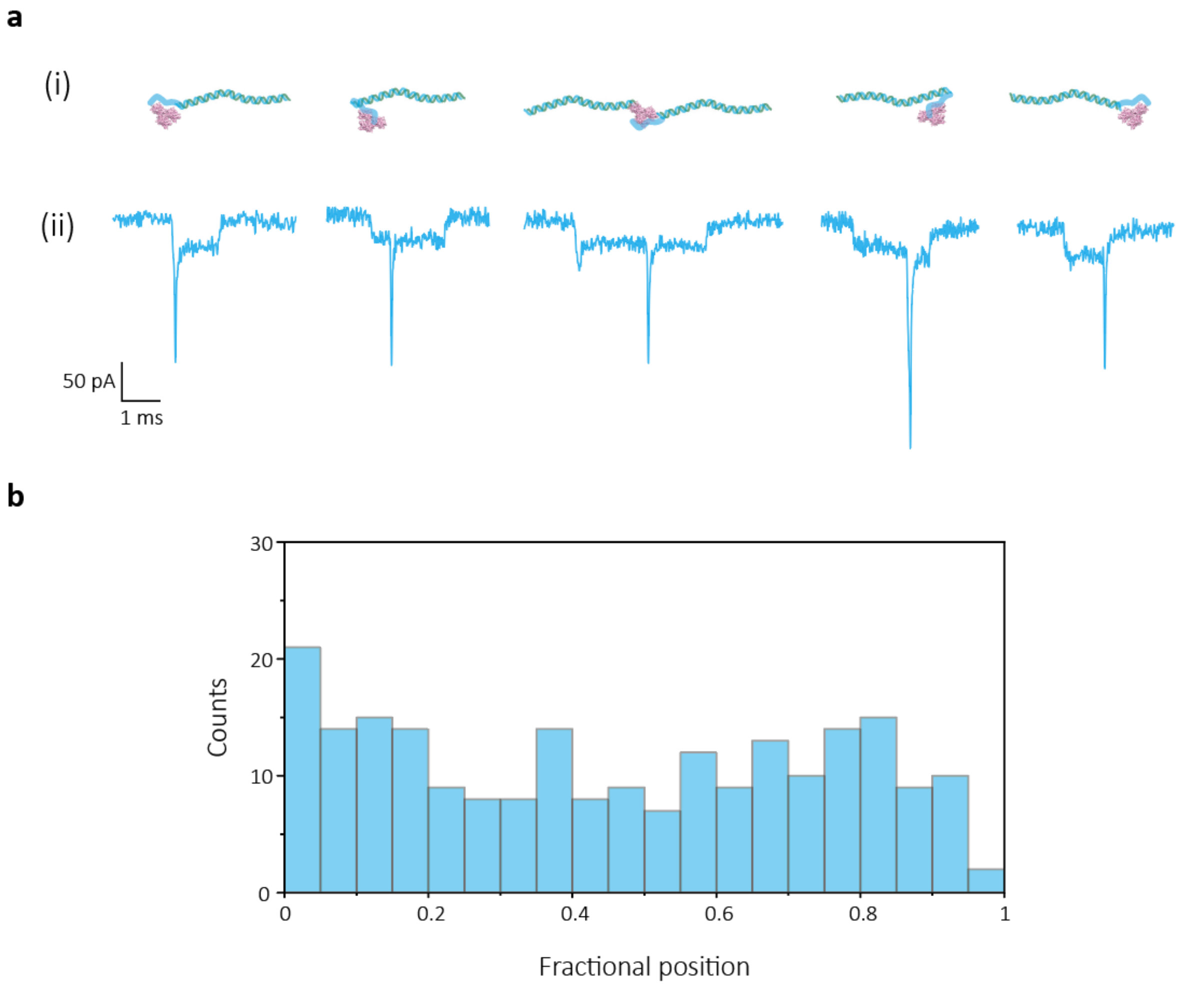

Figure S8. The fractional position for the subpeaks. (a) Schematic representations of different possible conformations of protein-bound carriers and their corresponding expected signals. **(b)** Fractional position analysis of the subpeaks observed for 6 h α-Syn oligomers bound to the carrier. The fractional position indicates the location of the subpeak within the total signal, where the beginning of the event is designated as '0' and the end as '1'. Variations in the subpeak position can be attributed to different binding orientations and conformations of the complexes as they translocate through the nanopore.

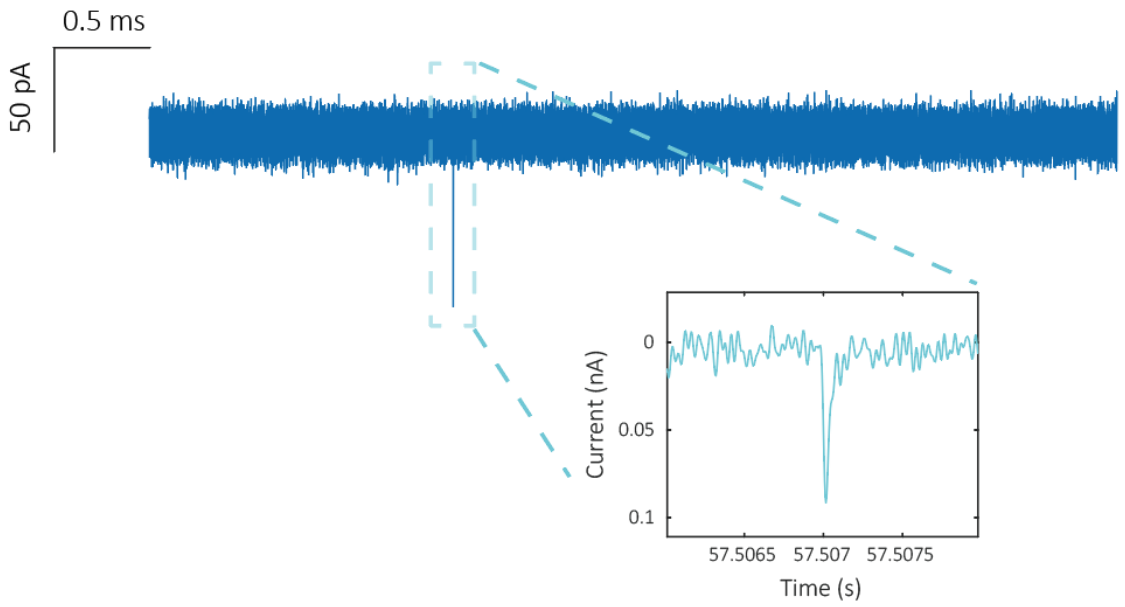

Figure S9. Nanopore detection of pure 6 h α-Syn oligomers. A typical 10-second current-time trace and a zoom-in window display a characteristic signal. The scale bar represents 0.5 ms horizontally and 50 pA vertically, respectively. The control experiment was conducted using 2 µM 6 h α-Syn oligomers in the same measuring buffer containing 2 M LiCl, 5 mM $MgCl_2$, and 10 mM Tris-EDTA (pH 8.0) at a voltage of + 300mV. We observed a very low capture rate of pure α-Syn samples, even using high concentrations; only 5 signals were detected during the recording of 30 min.

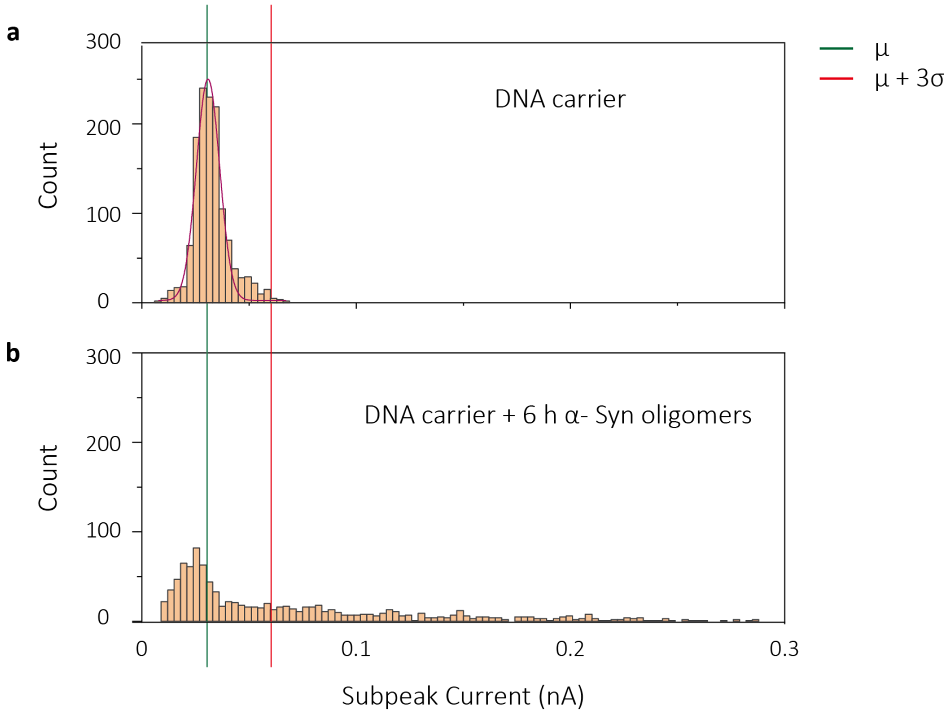

Figure S10. Thresholding of protein-bound subpeaks. Distributions of the subpeak current for **(a)** carrier only and **(b)** carrier bound with 6 h α-Syn oligomers. The mean and standard deviation of peak current for carriers was 34.2 ± 8.4 pA, based on the unfolded DNA carrier peak current. Using a threshold of mean plus three standard deviations, we could effectively exclude false positive signals resulting from partial DNA folding and accurately extract the protein-binding ratios for further analysis.

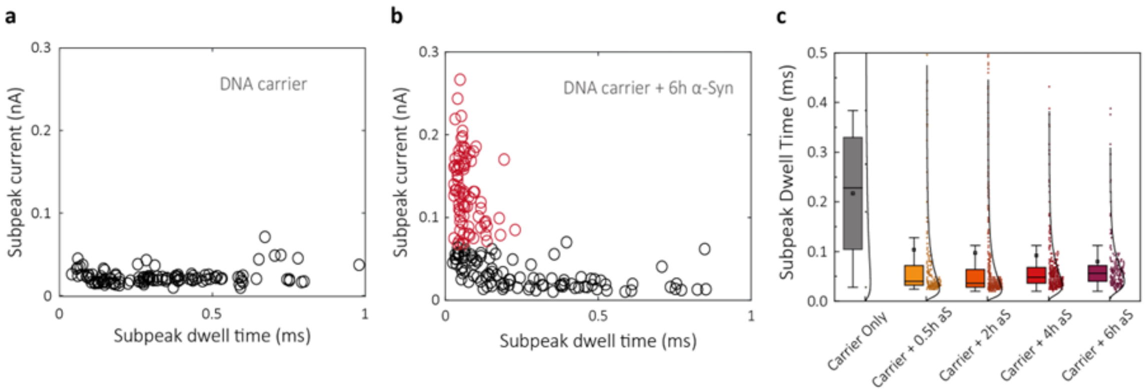

Figure S11. Subpeak analysis of protein-bound events. Scatter plots of subpeak current versus subpeak dwell time for **(a)** carrier only and **(b)** carrier bound with 6 h α-Syn oligomers. In these plots, the black circles represent the signals caused by DNA folding, while the red circles represent the signals caused by protein binding. In subpeak analysis of the carrier only, we can observe a relatively broad distribution in subpeak dwell time with low subpeak current; however, for carriers bound with α-Syn oligomers, a distinct population emerges, exhibiting a narrower distribution in subpeak dwell time with higher subpeak current. **(c)** Box and whisker plots of subpeak dwell time for carrier only and carriers bound with α-Syn oligomers collected from different aggregation times. Signals that exceed the predetermined threshold ($\mu \pm 3\sigma$) are selected for this analysis. The protein-bound carriers exhibited a decrease in the mean subpeak dwell time over the aggregation time, which can be attributed to the formation of larger oligomers that bind to the carriers. These highly negatively charged oligomers demonstrated faster nanopore translocation, leading to shorter subpeak dwell times.

Table S1. Clinical characteristics of Parkinson's disease and healthy control participants.

	PD1	PD2	PD3	PD4	PD5
Age, years	89	78	67	84	83
Sex	Female	Male	Male	Female	Female
Duration of Disease, years	7	8	8	13	11
Age of Onset, years	82	70	59	71	72
Clinical cause of death	PD dementia	PD, Broncho-pneumonia	PD, MSA	PD, Broncho-pneumonia	PD, chronic kidney disease

	HC1	HC2	HC3	HC4	HC5
Age, years	91	82	83	70	73
Sex	Female	Female	Male	Male	Female
Duration of Disease, years	N/A	N/A	N/A	N/A	N/A

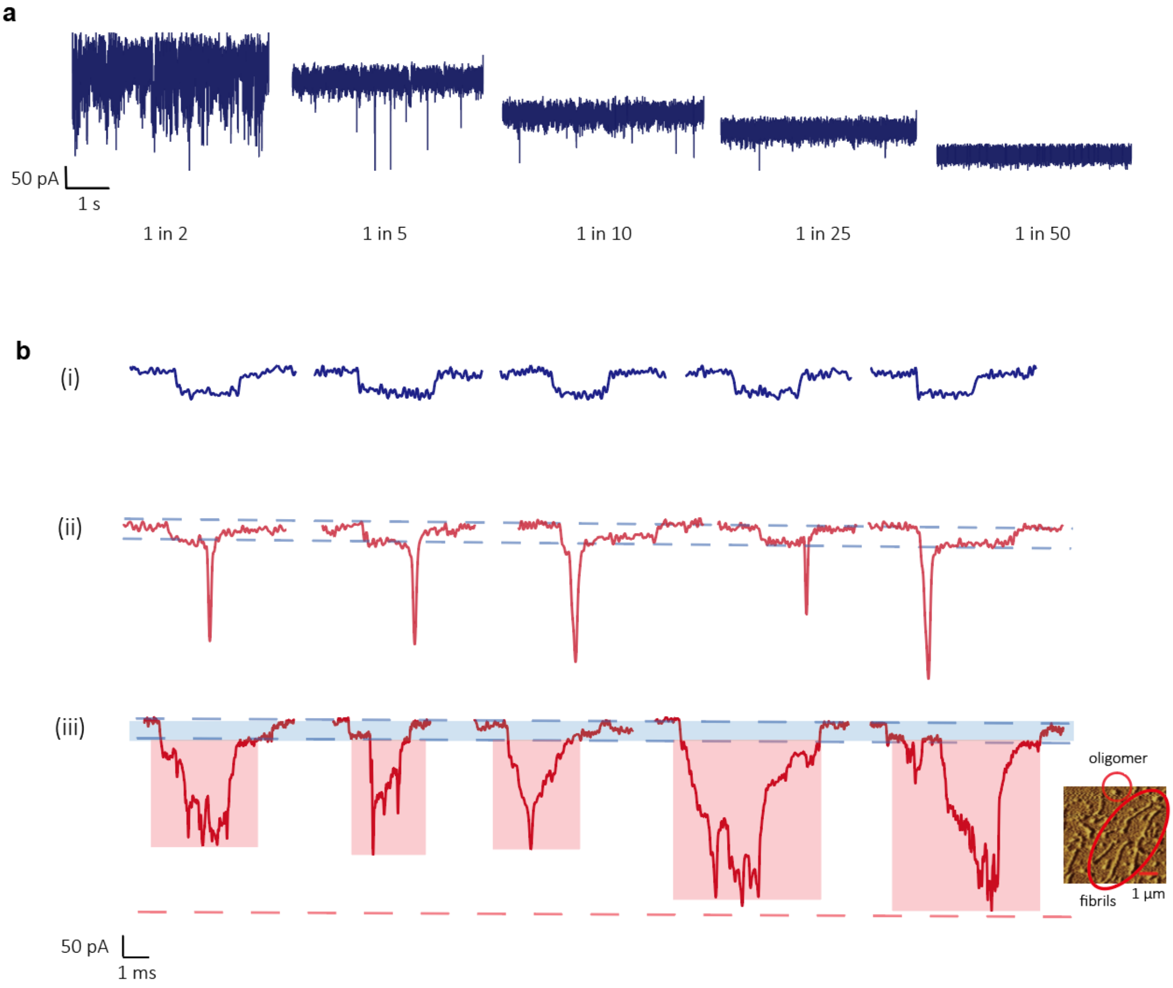

Figure S12. Nanopore detection of α-Syn oligomers in real CSF samples. (a) Typical traces for different CSF concentrations were diluted in the measurement buffer. The traces were measured using a series of dilutions from 1: 2 to 1: 50 (CSF: measuring buffer, v: v) at a voltage of + 300 mV. The optimal dilution with a low noise level was determined to be 1:50 for further measurements. **(b)** Typical signals of nanopore translocations in real CSF samples. (i) Signals obtained from the carriers in the healthy control samples, (ii) Signals obtained from the carriers bound with α-Syn oligomers in the patient samples, and (iii) Signals obtained from the carriers bound with α-Syn fibrils in the patient samples. As we analyzed the real patient samples, some signals shown in (iii) were observed with higher subpeak current and very long subpeak dwell time compared to previous results. We speculate this is due to the binding of the larger aggregates or fibrils formed in the patient's CSF. Inset: an AFM image confirming the presence of large aggregates and fibrils in the sample.

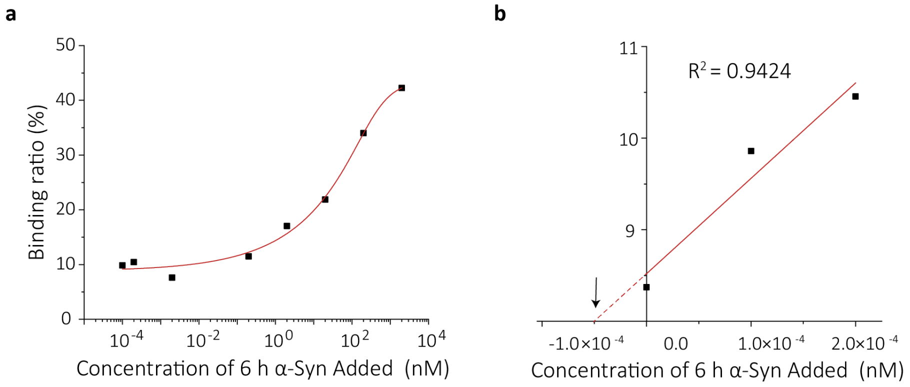

Figure S13. Standard addition assay of α-Syn oligomers in real CSF samples. Experiments were performed by adding a series of known concentrations of 6 h α-Syn aggregates in a diluted CSF solution (1: 50, CSF: buffer) from a healthy control (HC1) containing 200 pM DNA carriers. (a) The addition of α-Syn aggregates with known concentrations results in a response with a sigmoid shape. (b) The background concentration of the α-Syn oligomers in this CSF sample was determined to be 0.8 pM by a linear fit in the low-concentration region.

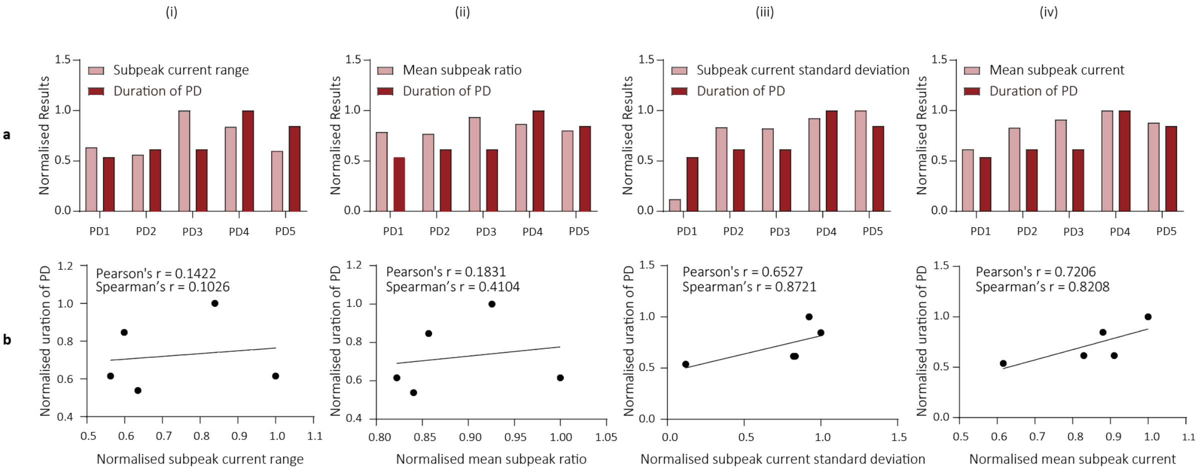

Figure S14. Correlation between nanopore readouts and duration of Parkinson's disease. (a) Histograms of the normalized results of nanopore readouts and the duration of PD for 5 PD patients. **(b)** Correlation analysis between normalized nanopore readouts and the duration of PD. We compared the results obtained from nanopore sensing with the actual duration of PD for 5 patients by evaluating four different methods: normalized (i) subpeak current range (maximum-minimum), (ii) mean subpeak ratio, (iii) standard deviation of subpeak current, and (iv) mean subpeak current. We accessed the correlation using Pearson's and Spearman's correlation coefficients. The Spearman's correlation coefficient assesses the strength and direction of a monotonic relationship between two results, and the relatively high Spearman's r value indicates that the relationship between the two results is consistent. The fourth method in b (iv), which yields the highest Pearson's r value and the second highest Spearman's value, suggests the strongest positive correlation.

Data Processing and Analysis

All recorded data was analyzed using a MATLAB App (The Nanopore App v.7.17) developed by Prof. Joshua Edel from Imperial College London.

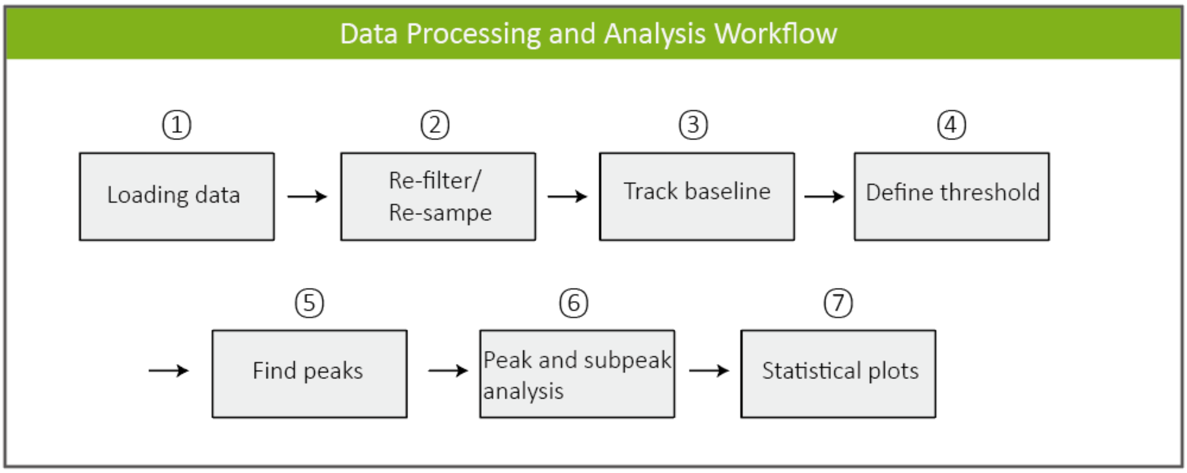

For the example shown below, data from 200 pM DNA diluted in patient CSF was analyzed. The experiment was conducted under a voltage of +300 mV.

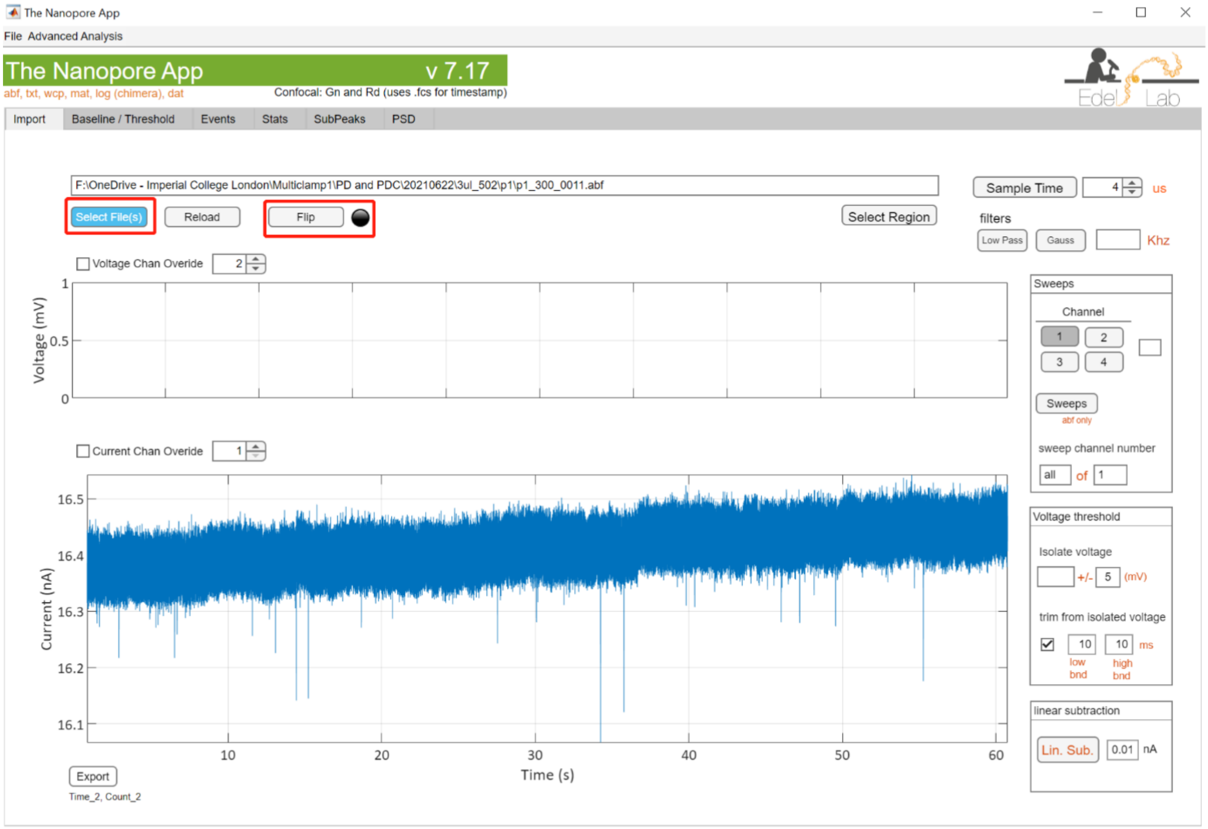

Figure 1. Loading data.

1. Loading the raw data. The Nanopore App supports several file formats, including ABF (Axon Digidata, Molecular Device), WCP (NI-DAQ, National Instruments), LOG, MAT (VC100, Chimera

Instruments), and DAT (eOne, Elements). To facilitate the data analysis the signal of the translocation events should point upwards. If it points downward as shown in Figure 1, the data should be flipped.

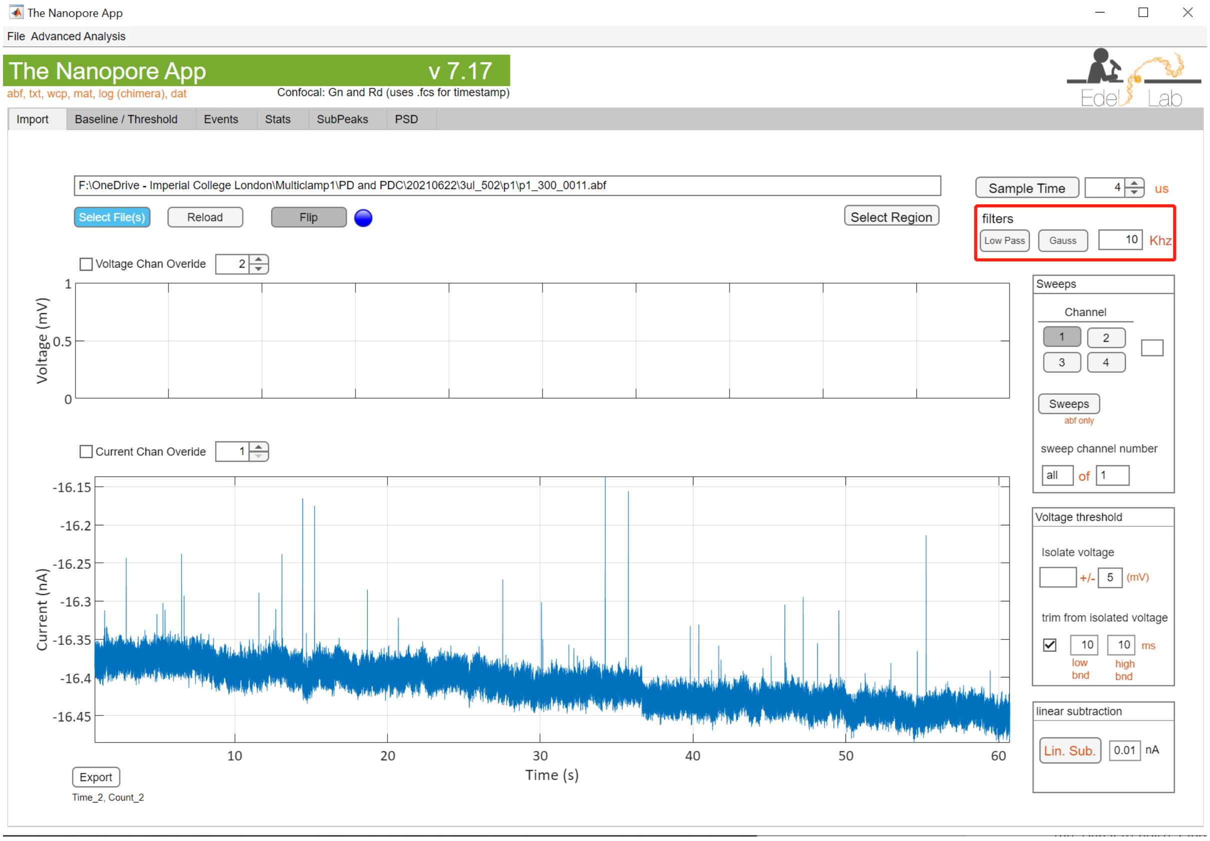

Figure 2. Refiltering the data.

2. <u>Re-sampling and re-filtering the data.</u> To enhance the clarity of the events, it is possible to perform resampling and refiltering. The original data were recorded at a high sampling rate and filtered (low-pass filter, 10 kHz).

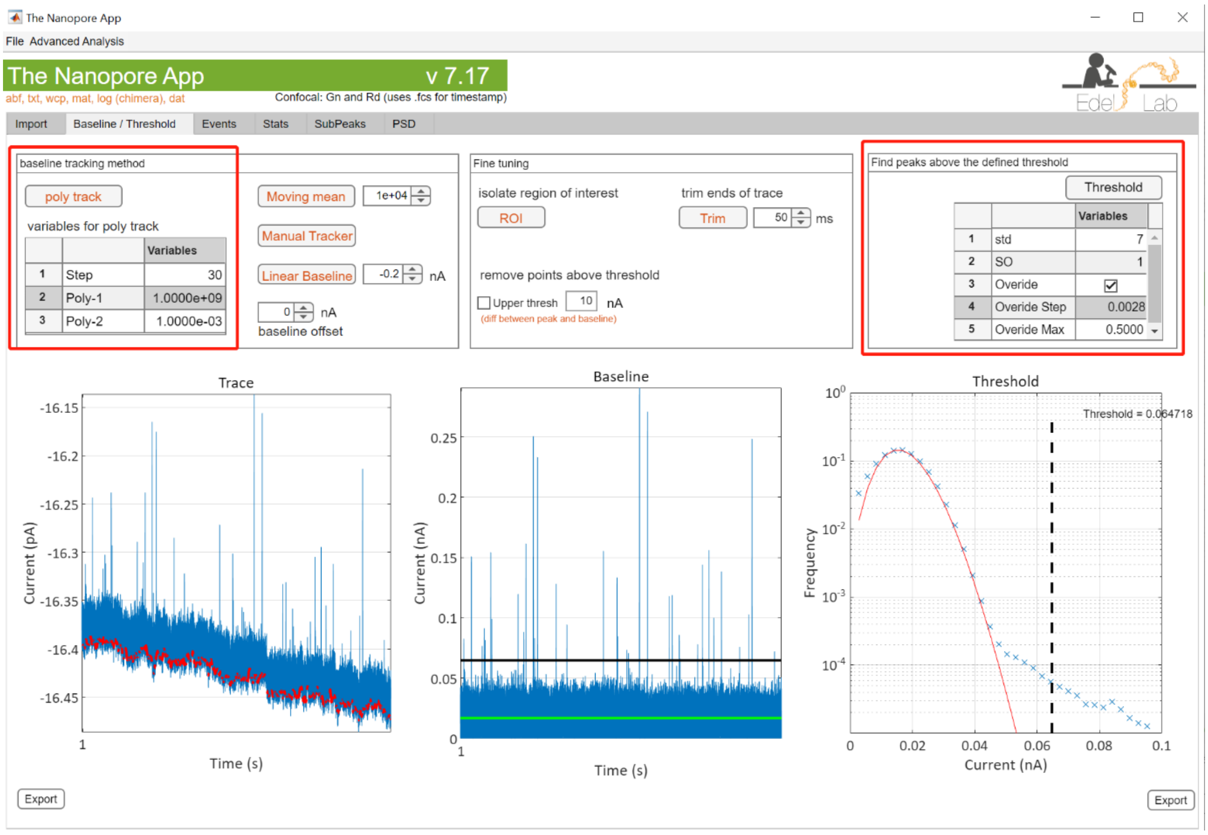

Figure 3. Baseline tracking and signal thresholding.

3. Tracking the baseline. To compensate for signal fluctuations and improve event classification, the baseline was tracked using a polynomial fit.

4. Defining a threshold. The signal was then histogrammed and fit to a Poisson distribution. This could then be used to determine an upper threshold (represented by the black line) based on a number of standard deviations above the mean.

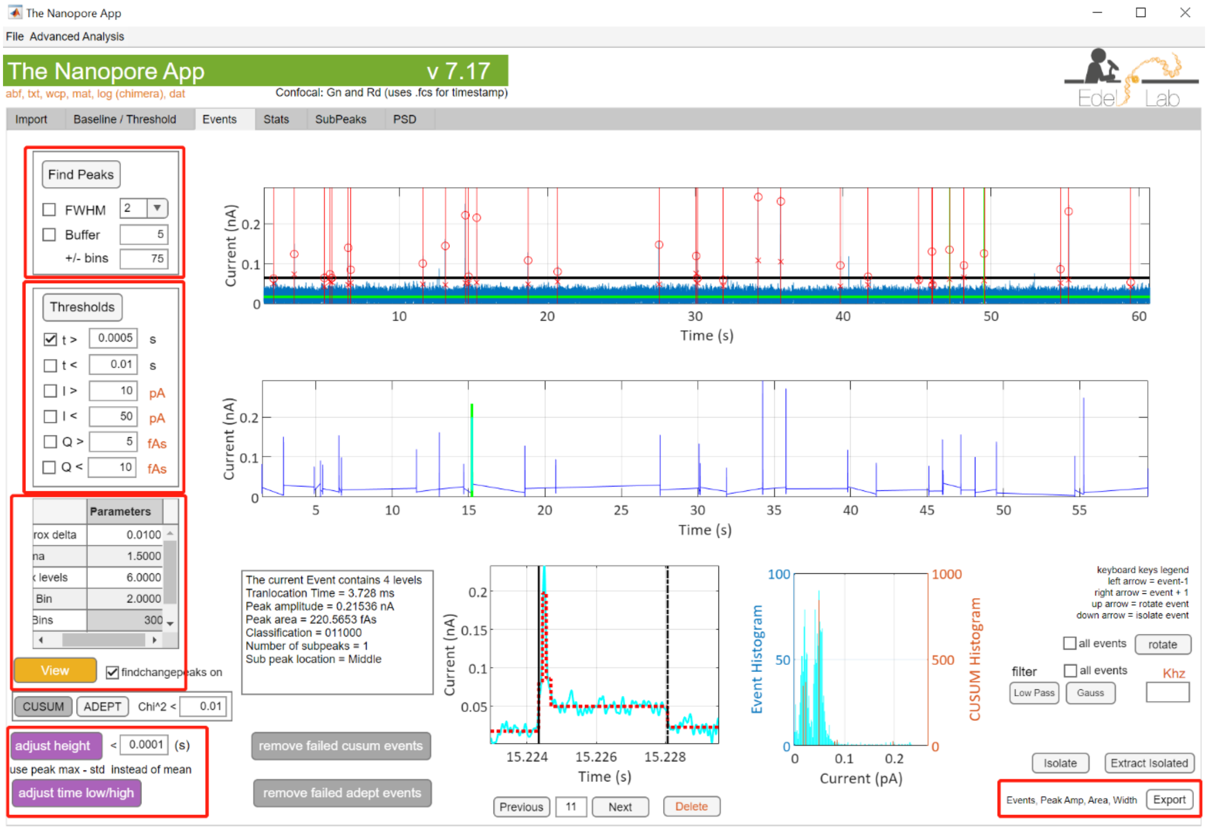

Figure 4. Peak finding.

5. Finding peaks. Based on the selected thresholds, events could be classified and isolated. Different thresholds, such as dwell time, peak current, and peak area, can also be applied as needed. This helps filter out noisy or irrelevant events, ensuring better event selection. A CUSUM fitting routine[1, 2] was used to fit each individual event. Information such as translocation time, peak amplitude, peak area, and subpeak information can be obtained as output parameters from the fit.

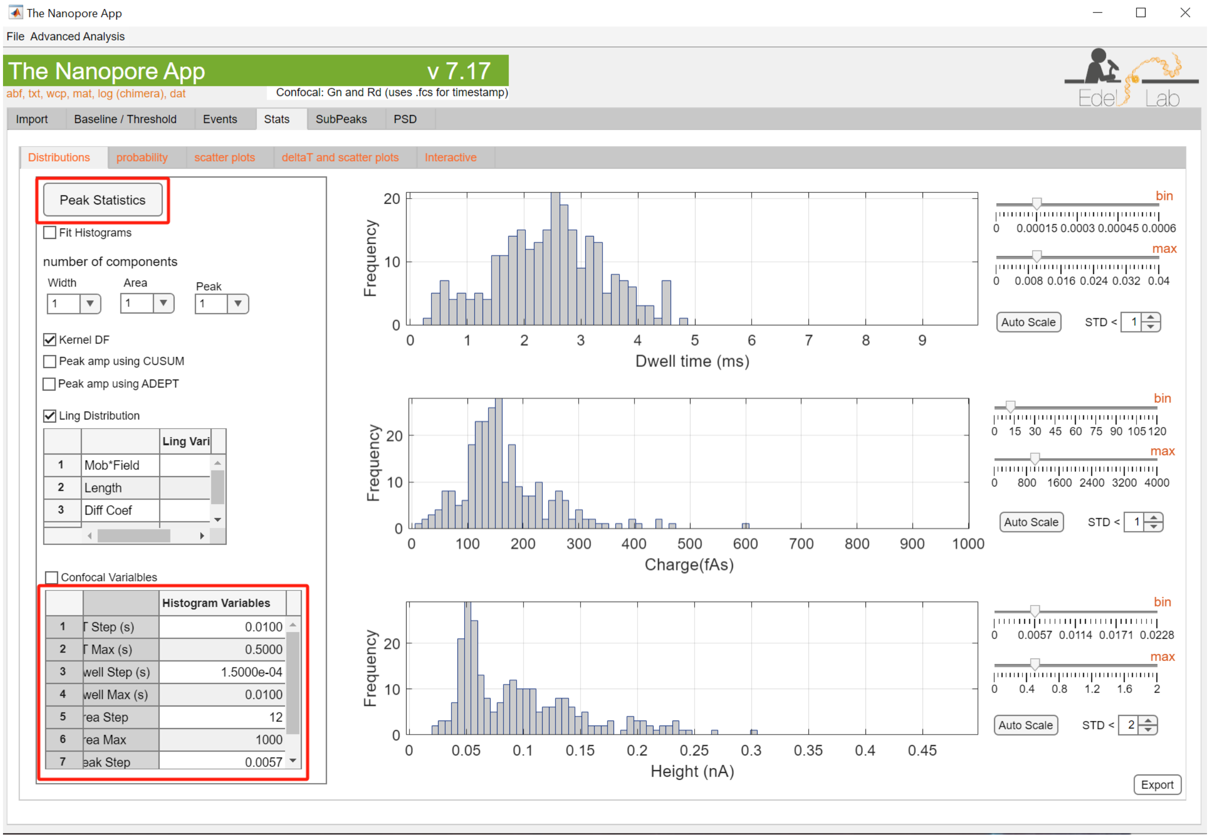

Figure 5. Peak statistics.

6. Peak statistics. The processed data underwent further statistical analysis to extract peak and subpeak information. The panel, depicted in Figure 5, provided direct observation and analysis of the distribution of dwell time, charge, and peak current results. The data can be easily exported by clicking the 'export' option to facilitate further analysis and generate high-quality figures.

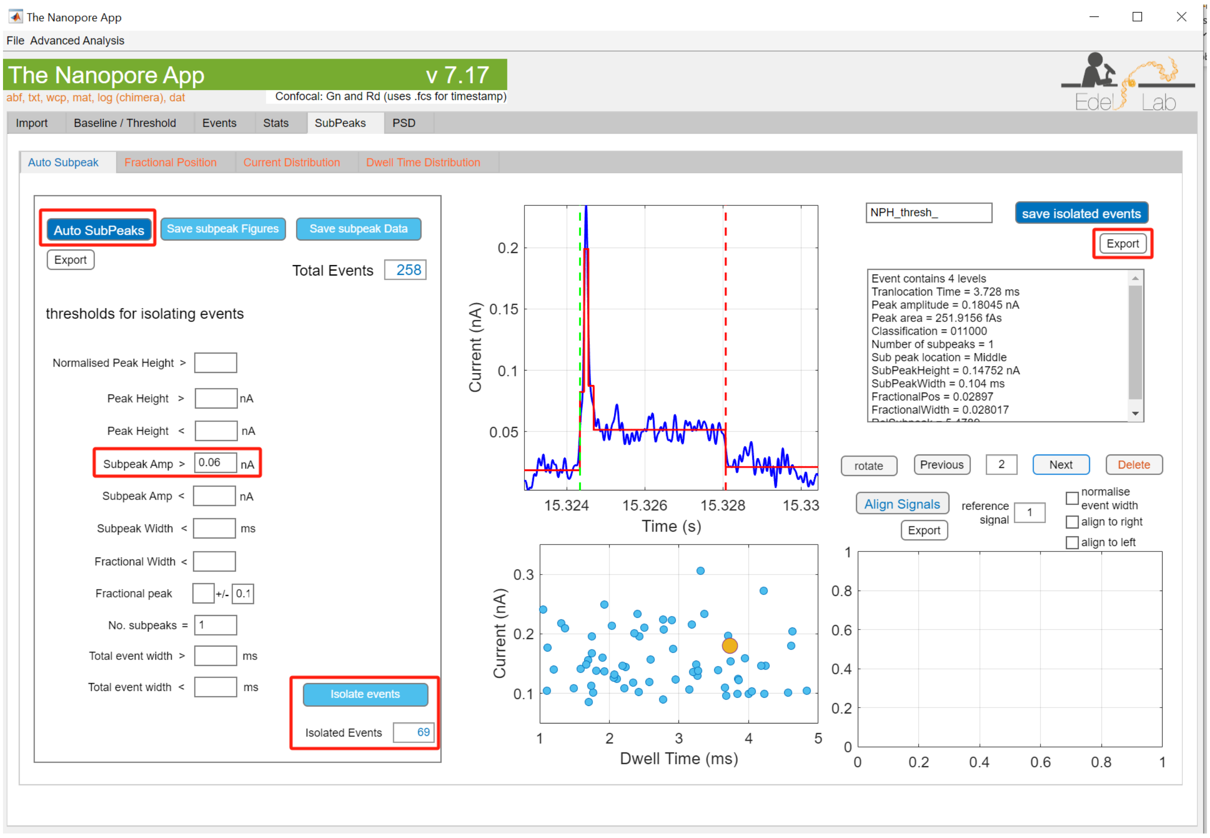

Figure 6. Subpeak analysis.

7. Subpeak statistics. The last step in the data analysis process involves extracting subpeak information. Selecting the 'Auto Subpeaks' option can directly retrieve crucial data from the CUSUM fits. This information includes subpeak amplitude, subpeak dwell time, fractional subpeak position, fractional subpeak width, the number of subpeaks, and their respective locations. These parameters are instrumental in distinguishing genuine positive events from potential false positives caused by DNA folding and can establish thresholds for this purpose. Fractional position is frequently used to isolate events associated with subpeaks originating from specific locations. Additionally, subpeak width, typically narrower for proteins than folded DNA, and peak amplitude can serve as discrimination criteria. 'Isolate events,' can visualize all events that meet the predefined thresholds. Moreover, the 'export' feature allows the extraction of additional information such as subpeak dwell time, subpeak current, subpeak position, and other pertinent data.

References

(1) Forstater, J. H.; Briggs, K.; Robertson, J. W. F.; Ettedgui, J.; Marie-Rose, O.; Vaz, C.; Kasianowicz, J. J.; Tabard-Cossa, V.; Balijepalli, A. MOSAIC: A Modular Single-Molecule Analysis Interface for Decoding Multistate Nanopore Data. *Anal. Chem.* **2016**, *88* (23), 11900-11907. DOI: 10.1021/acs.analchem.6b03725.

(2) Raillon, C.; Granjon, P.; Graf, M.; Steinbock, L. J.; Radenovic, A. Fast and automatic processing of multi-level events in nanopore translocation experiments. *Nanoscale* **2012**, *4* (16), 4916-4924, 10.1039/C2NR30951C. DOI: 10.1039/C2NR30951C.
